# Caucasian *Gentiana* Species: Untargeted LC-MS Metabolic Profiling, Antioxidant and Digestive Enzyme Inhibiting Activity of Six Plants

**DOI:** 10.3390/metabo9110271

**Published:** 2019-11-07

**Authors:** Daniil N. Olennikov, Aydan I. Gadimli, Javanshir I. Isaev, Nina I. Kashchenko, Alexey S. Prokopyev, Tatyana N. Kataeva, Nadezhda K. Chirikova, Cecile Vennos

**Affiliations:** 1 Laboratory of Medical and Biological Research, Institute of General and Experimental Biology, Siberian Division, Russian Academy of Science, 6 Sakh’yanovoy Street, 670047 Ulan-Ude, Russia; ninkk@mail.ru; 2 Department of Pharmacognosy, Azerbaijan Medical University, Anvar Gasimzade Street 14, Baku AZ1022, Azerbaijan; aydangadimli25@gmail.com (A.I.G.); isayev.cavanshir@amu.edu.az (J.I.I.); 3 Siberian Botanic Garden, Tomsk State University, Lenin Avenue 34/1, 634050 Tomsk, Russia; rareplants@list.ru (A.S.P.); gentianka@mail.ru (T.N.K.); 4 Department of Biochemistry and Biotechnology, North-Eastern Federal University, 58 Belinsky Street, 677027 Yakutsk, Russia; hofnung@mail.ru; 5 Regulatory and Medical Scientific Affairs, Padma AG, 30 Haldenstrasse, CH-8620 Wetzikon, Switzerland; c.vennos@padma.ch

**Keywords:** Gentiana, LC-MS profile, iridoid glycosides, flavone glycosides, xanthones, antioxidant activity, amylase/glycosidase inhibition

## Abstract

The members of *Gentiana* genus are widely distributed in the Caucasus region where they are used as phytoremedies, but they still have not been studied for their chemical composition and bioactivity. High-performance liquid chromatography with diode array and electrospray triple quadrupole mass detection (HPLC-DAD-ESI-QQQ-MS) was used to investigate metabolites of herb and roots of six gentians (*Gentiana asclepiadea*, *G. cruciata*, *G. gelida*, *G. paradoxa*, *G. pneumonanthe*, *G. septemfida*) grown in the Caucasus. In total, 137 compounds were found including three carbohydrates, 71 iridoid glycosides (mostly loganic acid), loganin, swertiamarin, gentiopicroside and sweroside derivatives, 40 flavones *C*-, *O*-, *C*,*O*-glycosides (such as luteolin, apigenin, chrysoeriol, and acacetin derivatives), two phenolic *O*-glycosides, five hydroxycinnamates, eight xanthones, and seven triterpene glycosides. Most of these compounds were identified in gentian samples for the first time. Quantitative differences were found in levels of seven iridoid glycosides, nine glycosylflavones, and two xanthones obtained by HPLC-DAD assay. The gentian extracts were evaluated for their radical-scavenging properties against DPPH and superoxide anion radicals, lipid peroxidation inhibition, and α-amylase/α-glycosidase inhibition. The herb extracts showed higher activity than root extracts. Positive correlations were found between the content of quantified phenolics and antioxidant and digestive enzymes inhibiting activity. The findings presented in our work suggest that the Caucasian gentians are a good source of bioactive phytocompounds with antioxidant and antidiabetic potential.

## 1. Introduction

**Figure 1:**
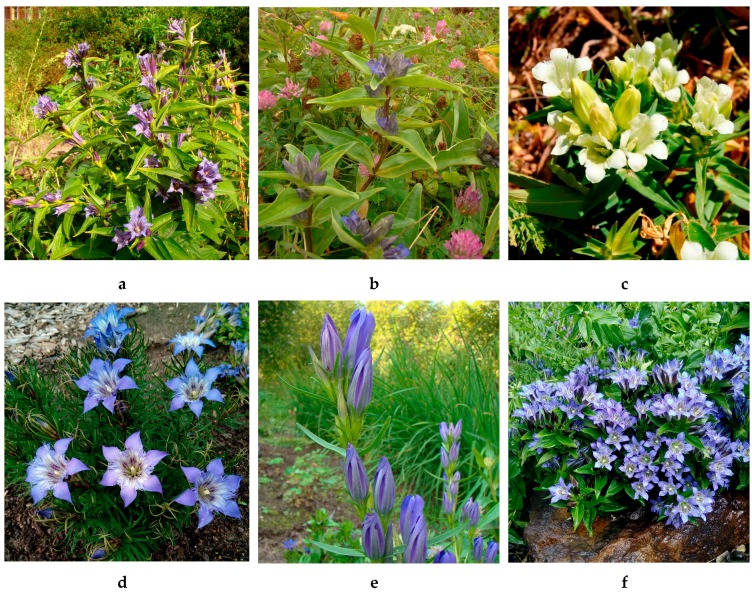
Caucasian gentians studied in present work: *Gentiana asclepiadea* (**a**), *G. cruciata* (**b**), *G. gelida* (**c**), *G. paradoxa* (**d**), *G. pneumonanthe* (**e**), *G. septemfida* (**f**).

*Gentiana* L. is a cosmopolitan gentianaceous genus involving about 360 species with a wide distribution in both hemispheres [1]. The diversity of forms and the broad range of ecological tolerance has allowed the gentians to adapt to the various natural conditions. As a rich phytogeographic region, the Caucasus was no exception and this region demonstrated the presence of about 20 species [2]. Many gentians were used in the traditional medical systems of the Caucasus native peoples. However, the latest published paper about medical plants of Caucasus often overlooked aspects of the gentians’ application in traditional medical practice despite their wide use by native peoples [3,4,5,6]. The known ethnopharmacological data on uses of the gentians as medical plants are various, and the plants have demonstrated a wide spectrum of pathology treatments (Supplementary Materials, Table S1). The most frequently used *Gentiana* species in the Caucasus region are *G. asclepiadea* (willow gentian), *G. cruciata* (star gentian), *G. gelida* (cold gentian), *G. paradoxa* (peculiar gentian), *G. pneumonanthe* (marsh gentian), and *G. septemfida* (crested gentian) (Figure 1). There are usual therapeutic recommendations for these species such as for appetizers, antipyretics, and antidiabetics. [7,8]. The various native peoples of Caucasus region also used mentioned species as remedies against hepatitis, anaemia, stomach pain, malaria, haemorrhoid, tuberculosis, bronchitis, and pneumonia [9,10,11].

Some chemical aspects of five species (*G. asclepiadea*, *G. cruciata*, *G. gelida*, *G. pneumonanthe*, *G. septemfida*) were shown previously, but *G. paradoxa* is still an unstudied species. The known data include information about flavonoids (flavones only) in four species [11,12,13,14,15,16,17,18,19,20,21,22,23,24,25], xanthones (mangiferin, gentisin and its glycosides) in three species [15,16,17,18,20,26,27], iridoid glycosides in five species [18,19,22,27,28,29,30,31,32], monoterpenes in *G. pneumonanthe* [32], and triterpenes and naphthodipyranodione in *G. asclepiadea* [32,33]. In total, thirty-nine compounds were found in five gentians with most diverse classes of flavonoids and iridoid glycosides (Table S2). We also establish some facts about pharmacological uses of *G. asclepiadea* and *G. cruciate,* and the spectrum of gentians’ bioactivity includes cytotoxic [34], antimicrobial [35], antigenotoxic [36], antioxidant [37], anticholinesterase [19], hepatoprotective [38], and antibiofilm potential [39]. Based on this short review, it is obvious that Caucasian gentians should receive more attention as sources of phytocompounds and bioactive plant remedies.

It is particularly significant that high-performance liquid chromatography (HPLC) profiling with diode array detection and/or mass detection was performed only for two species, (*G. asclepiadea* [37], *G. cruciata* [22]), although in limited manner. Therefore, a comprehensive and comparative study is needed for a clear understanding of the chemo-diversity of Caucasian gentians. The antioxidant studies of gentians are for the same reason and also essential to identify antioxidant principles of plants. The information about antidiabetic activity of selected gentians is still unknown; so, it would be useful to know their potential against key enzymes of carbohydrate metabolism such as α-amylase and α-glycosidase [40]. The aim of present paper was to profile soluble metabolites of six gentian herbs and roots using high-performance liquid chromatography with diode array and electrospray triple quadrupole mass detection (HPLC-DAD-ESI-QQQ-MS) techniques and to quantify selected flavonoids, xanthones and iridoid glycosides in gentian plants. In this paper, we also make a comparative study of antioxidant activity and the digestive enzyme inhibition potential of gentian extracts, and we found active compounds which were the bioactive principles of the gentians.

## 2. Results and Discussion

### 2.1. Liquid Chromatography Mass Spectrometric (LC-MS) Metabolite Profiling of Six Caucasian Gentians: Chemodiversity of Herbs and Roots

An assay based on high-performance liquid chromatography with diode array and electrospray triple quadrupole mass detection (HPLC-DAD-ESI-QQQ-MS) was used to profile soluble metabolites of the herbs and roots of *Gentiana asclepiadea*, *G. cruciata*, *G. gelida*, *G. paradoxa*, *G. pneumonanthe*, and *G. septemfida* collected in Caucasus. A comparison of the ultraviolet (UV) spectra, mass spectral with daughter fragmentation (MS^n^) data, and retention times (Figure S1) with reference compounds (Figure S2) and literature data were used for identification of compounds (Table S3). The chromatograms demonstrated the presence of 137 compounds in six gentian herbs and roots (Table 1).

#### 2.1.1. Carbohydrates

Highly hydrophilic components of gentian herbs and roots eluted with retention times 2.78–3.17 min were carbohydrates such as hexose (**4**; *m*/*z* 179 [M–H]^–^), *O*-hexosyl-hexose (**2**; *m*/*z* 341 [M–H]^–^), and *O*-hexosyl-*O*-hexosyl-hexose (**3**; *m*/*z* 503 [M–H]^–^). Typical monosaccharides of gentians are glucose and fructose, disaccharides – gentiobiose and saccharose, and trisaccharides – gentianose and gentiotriose [41]. The limitation of the RP-HPLC assay used is poor separation of isomeric carbohydrates. Therefore, an additional study is needed to clarify the carbohydrate profile of the gentians.

#### 2.1.2. Iridoid Glycosides

Iridoid glycosides were the most diverse group of metabolites and it includes 71 compounds. The following types of iridoid glycosides were detected in studied gentians.

**Figure 2:**
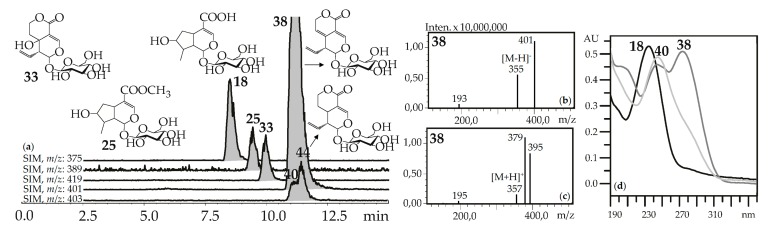
(**a**) HPLC-ESI-MS chromatogram of *G. septemfida* root extract in SIM-mode {*m*/*z* 375 for loganic acid (**18**), *m*/*z* 389 for loganin (**25**), *m*/*z* 419 for swertiamarin (**33**), *m*/*z* 401 for gentiopicroside (**38**), *m*/*z* 403 for sweroside (**40;** isomeric **44**)}. (**b**,**c**) Mass spectra of compound **38** in negative and positive ionization mode. (**d**) UV spectra of compounds **18**, **38** and **40**.

“Usual” iridoid glycosides consisted of iridoid aglycone and a hexose (glucose) fragment with typical mass spectrometric patterns in negative ionization mode that included an intense signal for the deprotonated ion [M–H]^–^ and/or an ion-adduct [(M–H)+HCOOH]^–^ and weak signal for the dehexosylated fragment [(M–H)–Hex]^−^ (Figure 2). The positive ionization mass spectra demonstrated the presence of weak signals from the protonated ion [M+H]^+^ and dehexosylated ion [(M+H)–Hex]^+^ and strong signals of adduct ions such as [M+Na]^+^ and/or [M+K]^+^. Loganic acid (**18**), loganin (**25**), swertiamarin (**33**), gentiopicroside (**38**), and sweroside (**40**) are the most common examples of these types of iridoid glycosides found in gentians here and elsewhere [42], and they were identified using standards.

The rare, for the gentians, morroniside (**23**) was also compared with a standard and found in the *G. septemfida* herb only. In addition to the mentioned iridoid glycosides, similar mass spectrometric patterns were found for twenty-one compounds. There were compounds isomeric to swertiamarin (**41**), gentiopicroside (**56**), and sweroside (**44**) and eighteen compounds with less obvious structures. Compound **14** gave a deprotonated ion with *m*/*z* 389 and was tentatively identified as eustomoside, an iridoid glycoside first found in *Eustoma russellianum* [43] and later in *G. septemfida* herb [28]. Seven compounds (**3**, **5**, **6**, **10**, **13**, **17**, **19**) have the molecular weight 408 and these could be isomers of eustomorusside detected in the *G. septemfida* herb [28]. A tentative identification was made for compound **12**, which gave a deprotonated ion with *m*/*z* 471 that is usual for eustoside, which was also discovered in the *G. septemfida* herb [28]. From the spectral data of compounds **7** (*m*/*z* 477 [M–H]^–^), **22**, **24** (*m*/*z* 445 [M–H]^–^), **67** (*m*/*z* 683 [M–H]^–^), **96** (*m*/*z* 435 [M–H]^–^), and **86**, **100** (*m*/*z* 561 [M–H]^–^), they were concluded to have an iridoid glycoside nature.

Iridoid glycosides with additional sugar fragments have molecular weights of 162 a.m.u. more than the parent compound. Also, in the MS^n^ spectra, they gave extra signals of dehexosylated fragments [(M–H)–*n*×Hex]^–^. Loganic acid-6′-*O*-glucoside (**15**) found in *G. cruciata* roots gave a deprotonated ion with *m*/*z* 537 and, in the MS^2^ spectrum, signals with *m*/*z* 375 and 213 belonging to the fragments [(M–H)–Glc]^–^ and [(M–H)–2×Glc]^–^, respectively. Similar spectral patterns were detected in the mass spectra of swertiamarin-6′-*O*-glucoside (**26**) and its isomers **8** and **9**, gentiopicroside-6′-*O*-glucoside (**27**) and gentiopicroside-di-*O*-hexoside (**20**), and sweroside-6′-*O*-glucoside (**28**).

Iridoid glycosides with a 2,3-dihydroxybenzoyl fragment were characterized by specific UV absorptions at 235–238, 255–257 and 322–326 nm, and the mass spectra demonstrated the loss of a fragment with 136 a.m.u. belonging to an aromatic acid [44]. These types of iridoids are rare in plants, and they are distributed mainly in the *Gentiana* genus [42]. Eight iridoid glycosides were found including the known compounds 2′-*O*-(2,3-dihydroxybenzoyl)-loganic acid (algidiside I, **58**), 6′-*O*-(2,3-dihydroxybenzoyl)-loganic acid (algidiside II, **69**) [14], and 6′-*O*-(2,3-dihydroxybenzoyl)-sweroside (**83**) [44]. Other dihydroxybenzoyl ethers with unknown types of substitution were derivatives of loganic acid (**77**), loganin (**91**, **103**), and gentiopicroside (**74**, **81**).

Iridoid glycosides with 2,3-dihydroxybenzoyl and acetyl fragments have spectral properties close to the previous group with additional signals in mass spectra generated by elimination of acetyl moieties (42 a.m.u.). Tri-*O*-acetyl-*O*-2,3-dihydroxybenzoyl-swertiamarin (**134**) and two tri-*O*-acetyl-*O*-2,3-dihydroxybenzoyl-swerosides **111** and **135** were found in four gentians (*G. asclepiadea*, *G. gelida*, *G. paradoxa*, *G. septemfida*) and their most likely structures were deglucosylated gelidosides (found previously in *G. robusta* [45]) and deglucosylated trifloroside, described in *Gentiana triflora* subsp*. japonica* (Kusn.) Vorosch. [46].

Iridoid glycosides with 2,3-dihydroxybenzoyl and hexose fragments were characterized by the primary loss of a hexosyl fragment with *m*/*z* 162 followed by the expected elimination of a 2,3-dihydroxybenzoyl moiety (*m*/*z* 136). Two loganic acid-*O*-2,3-dihydroxybenzoyl ether-*O*-hexosides, **59** and **62,** from *G. asclepiadea* roots and sweroside-*O*-2,3-dihydroxybenzoyl ether-*O*-hexoside, **72,** from *G. septemfida* roots were detected. The latter compound’s structure could be tentatively identified as a deacetylated trifloroside compound. A similar structure was found in *G. straminea* roots and identified as 6′-*O*-{(2″-hydroxy-3″-glucosyloxy) benzoyl}-sweroside [47], also known as a gentiotrifloroside isolated from *G. triflora* [48]. Compounds **59** and **62** have no analogues in plants. 

**Figure 3:**
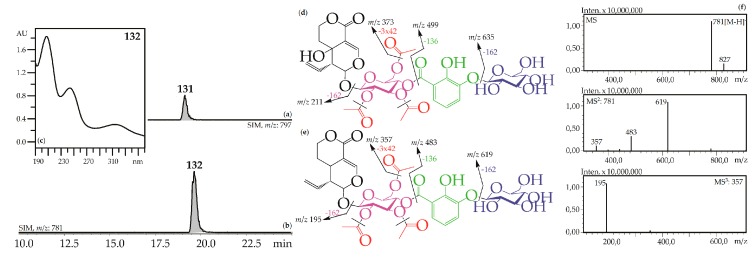
(**a,b**) HPLC-ESI-MS chromatogram of *G. gelida* root extract in SIM-mode: {*m*/*z* 797 for gelidoside (rindoside, **131**, **a**); *m*/*z* 781 for trifloroside (**132**, **b**)}. (**c**) UV spectrum of compounds **132**. (**d,e**) Mass spectrometric fragmentation of compounds **131** (**d**) and **132** (**e**). (**f**) MS^n^ spectra of compound **132**.

Iridoid glycosides with 2,3-dihydroxybenzoyl, acetyl and hexose fragments were members of the largest group of iridoids found in Caucasian gentians. Their mass spectra contain a sequence of signals caused by the serial elimination of hexose (or hexoses), a 2,3-dihydroxybenzoyl fragment, an acetyl group (or groups), and final loss of glucose and liberation of an iridoid aglycone (Figure 3). Twenty-one compounds gave an analogous fragmentation pattern. Among these were derivatives of loganic acid (**82**, **89**, **130**), swertiamarin (**93**, **102**, **108**, **113**, **117**, **120**, **128**, **131**), sweroside (**101**, **110**, **121**, **126**, **127**, **129**, **132**) and tentatively eustomorusside (**88**) and eustomoside (**116**, **133**). Comparison with standard compounds allowed identification of gelidoside (rindoside, **131**) and trifloroside (**132**) which were 2′,3′,6′-tri-*O*-acetyl-4′-*O*-{(2″-hydroxy-3″-glucosyloxy) benzoyl}-swertiamarin [29] and 2′,3′,6′-tri-*O*-acetyl-4′-*O*-{(2″-hydroxy-3″-glucosyloxy) benzoyl}-sweroside, respectively [46]. When replacing swertiamarin in **131** or sweroside in **132** with loganic acid, the new iridoid glycosides **82**, **89**, and **130** would be obtained, but real examples of this are still unknown.

Three monoacetylated (**93**, **102**, **108**) and two diacetylated (**113**, **117**) analogues of gelidoside were found in *G. gelida*, *G. cruciata*, *G. pneumonanthe* and *G. septemfida* as well as two hexosylated derivatives of gelidoside (**120**, **128**). Only one diacetylated compound, 3′,4′-di-*O*-acetyl-6′-*O*-{(2″-hydroxy-3″-*O*-glucopyranosyloxy)- benzoyloxy}-swertiamarin (gentistraminoside B), was found previously as a component of *G. straminea* roots [49]. Data about monoacetylated and hexosylated gelidosides is absent. Two compounds with one acetyl group (**101**, **110**), two compounds with two acetyl groups (**121**, **132**), and two hexosylated derivatives of trifloroside (**126**, **129**) were discovered in gentians. The known data about similar compounds include two monoacetylated analogues of trifloroside – 3′-*O*-{(2″-hydroxy-3″-glucosyloxy) benzoyl}-6′-*O*-acetyl-sweroside from *G. manshurica* roots [50] and 4′-*O*-acetyl-6′-*O*-{(2″-hydroxy-3″-glucosyloxy)benzoyl}-sweroside (gentistraminoside A) from *G. straminea* roots [49], and two hexosylated triflorosides such as 4‴-*O*-glucosyl trifloroside from *G. scabra* roots [51] and 6′"-*O*-glucosyl trifloroside from *G. linearis* roots [52]. The unknown compound, which was found in *G. gelida* herb and gave a deprotonated molecular ion with *m*/*z* 831, was tentatively identified as tri-*O*-acetyl-*O*-2,3-dihydroxybenzoyl-*O*-hexosyl-eustomorusside, **88**. Compounds **116** and **133** were two isomeric tri-*O*-acetyl-*O*-2,3-dihydroxybenzoyl-*O*-hexosyl-eustomosides for which only one known structure is available, 2′,3′,6′-tri-*O*-acetyl-4′-*O*-{(2″-hydroxy-3″-glucosyloxy) benzoyl}- eustomoside or gentomoside isolated from *G. gelida* [29].

Iridoid glycosides with a caffeoyl fragment have specific UV patterns with maxima at 325–330 nm, and their mass spectra gave the signals of decaffeoylated ions derived from the parent molecular ions after loss of a fragment with *m*/*z* 162 (caffeoyl). Two compounds were found and identified as *O*-caffeoyl-loganic acid (**76**) from *G. gelida* roots and *O*-caffeoyl-sweroside (**115**) from *G. cruciata* roots. The known iridoid glycoside caffeates are 2′-*O*-caffeoyl-loganic acid from *G. loureirii* herb [53] and 3′-*O*-caffeoyl-sweroside from *Anthocephalus chinensis* bark (Rubiaceae) [54].

One compound, amarogentin (**105**), was not among the mentioned types of gentian iridoid glycosides and was identified using a standard compound. Amarogentin is a common bitter component of *G. lutea* roots and was also found to be a trace compound in the roots of *G. asclepiadea* and *G. pneumonanthe* [27]. In our study it was detected only in *G. gelida* roots.

#### 2.1.3. Phenolic Acid O-glycosides

**Figure 4:**
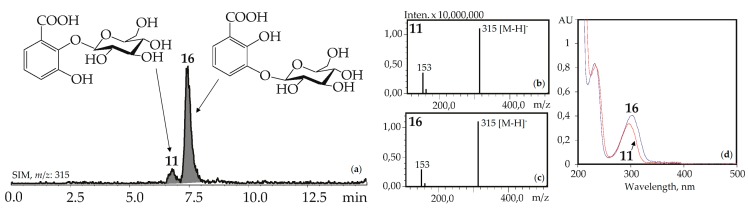
(**a**) HPLC-ESI-MS chromatogram of *G. gelida* herb extract in SIM-mode (*m*/*z* 315). (**b**, **c**) Mass spectra (negative ionization) of compounds **11** (2,3-dihydroxybenzoic acid 2-*O*-glucoside) and **16** (2,3-dihydroxybenzoic acid 3-*O*-glucoside), respectively. (**d**) UV spectra of compounds **11** and **16**.

The UV patterns of two compounds, **11** and **16,** were close (λ_max_ 203, 235–240, 300–304 nm) and specific for the 2,3-dihydroxybenzoic acids with one substituted hydroxyl (Figure 4). Basic fragments in the negative mass spectra of **11** and **16** showed signals with *m*/*z* 315 [M–H]^–^ and 153 [(M–H)–hexose]^–^ that are characteristic for *O*-hexosides of 2,3-dihydroxybenzoic acid [55]. The most likely identification of the dominant **16** is 2,3-dihydroxybenzoic acid 3-*O*-glucoside, because its fragment can be seen in the structures of gelidoside (rindoside, **131**) and trifloroside (**132**), suggesting a need of **16** for biosynthesis of selected iridoids in gentians. The trace component **11** is isomeric to **16** and may be discovered as 2,3-dihydroxybenzoic acid 2-*O*-glucoside. Both compounds were found in gentians for the first time. 

#### 2.1.4. Hydroxycinnamates

Hydroxycinnamates are rare gentian components. Only five caffeic acid derivatives were detected in two gentians including 1-*O*-caffeoyl-glucose (**21**) and 6-*O*-caffeoyl-glucose (**45**) from *G. pneumonanthe* herb and 2-*O*-caffeoyl-glucaric acid (**51**), 1,3-di-*O*-caffeoyl-glycerol (**79**), and 1,2-di-*O*-caffeoyl-glycerol (tentative, **90**) from *G. septemfida* herb and none of these have been found in gentians. Previously known gentian hydroxycinnamates are ferulic acid in *G. scabra* roots, *O*-feruloyl-glucose from *G. loureirii* whole plant, and 3-*O*-caffeoyl-glucose from *G. rigescens* roots [42].

#### 2.1.5. Xanthones

**Figure 5:**
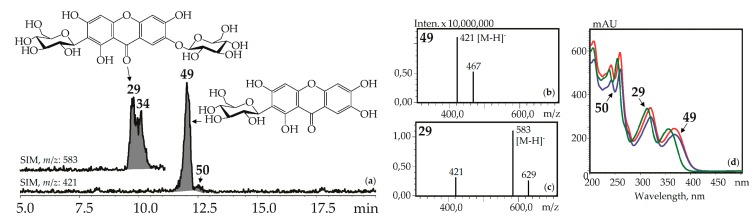
(**a**) HPLC-ESI-MS chromatogram of *G. asclepiadea* herb extract in SIM-mode (*m*/*z* 421, 583). (**b**, **c**) Mass spectra (negative ionization) of compounds **49** (mangiferin) and **29** (mangiferin-7-*O*-glucoside), respectively. (**d**) UV spectra of compounds **29, 49,** and **50**.

Xanthone-*C*-glycosides mangiferin (1,3,6,7-tetrahydroxyxanthone-2-*C*-glucoside, **49**) and isomangiferin (1,3,6,7-tetrahydroxyxanthone-4-*C*-glucoside, **50**) were identified using reference compounds, UV spectra (λ_max_ 240, 257, 318, 365 nm), and mass spectrometric patterns (*m*/*z* 421 [M–H]^–^, 467 [(M–H)+HCOOH]^–^). These were in known sources such as the herbs of *G. asclepiadea* [15], *G. cruciata* [17], *G. pneumonanthe* [26], and for the first time in *G. paradoxa* herb (Figure 5). 

Additionally, two isomeric to mangiferin compounds, **30** and **39,** with lower retention times were found in *G. cruciata* and *G. paradoxa*. Mangiferin-7-*O*-glucoside (neomangiferin, **29**) gave the signals of deprotonated ion [M–H]^–^ with *m*/*z* 583 and a deglucosylated fragment with *m*/*z* 421 [(M–H)–glucose]^–^. It is a known component of *G. asclepiadea* herb [20] and was newly found in *G. cruciata*, *G. paradoxa*, and *G. pneumonanthe*. A compound isolated from *G. asclepiadea* herb, but with a longer retention time than **29**, was tentatively identified as mangiferin-6-*O*-glucoside, **34**, an isomer of **29** [20]. Gentisin (1,7-dihydroxy-3-methoxyxanthone, **137**) and its 1-*O*-primveroside (gentioside, **109**) were identified using reference compounds as a component of *G. asclepiadea* roots.

#### 2.1.6. Flavonoids

Forty *O*-, *C*-, and *O*,*C*-glycosides and acylated compounds were identified as derivatives of luteolin, apigenin, chrysoeriol, and acacetin. Only one aglycone chrysoeriol (**136**) was detected in herbs of *G. asclepiadea* and *G. pneumonanthe*.

Among the fifteen luteolin glycosides found, seven compounds compared with reference compounds were luteolin-7-*O*-glucoside (**98**), luteolin-6-*C*-glucoside (isoorientin, **61**), luteolin-8-*C*-glucoside (orientin, **80**), isoorientin-7-*O*-glucoside (**35**), isoorientin-2″-*O*-glucoside (**43**), isoorientin-4″-*O*-glucoside (**52**), and isoorientin-6″-*O*-glucoside (**54**). Isoorientin is one of the most often described flavonoids of the *Gentiana* genus and it was found in all herb samples and in *G. asclepiadea* root. The same distribution was shown for the isoorientin-2″-*O*-glucoside, the known flavonoid of *G. asclepiadea* herb [15]. Compound **63** was isomeric to **35**, **43**, **52**, and **54** and was determined as luteolin-*C*-hexoside-*O*-hexoside.

**Figure 6:**
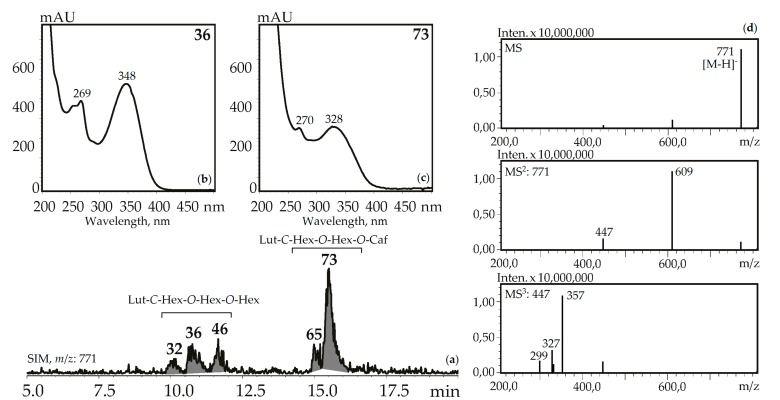
(**a**) HPLC-ESI-MS chromatogram of *G. gelida* herb extract in SIM-mode (*m*/*z* 771). (**b**, **c**) UV spectra of compounds **36** and **73**. (**d**) MS^n^ spectra (negative ionization) of compound **36**. Lut-*C*-Hex-*O*-Hex-*O*-Hex—zone of isomeric luteolin-*C*-hexoside-*O*-hexoside-*O*-hexosides, Lut-*C*-Hex-*O*-Hex-*O*-Caf—zone of isomeric luteolin-*C*-hexoside-*O*-hexoside-*O*-caffeates.

The UV spectrum of compound **97** was specific for the luteolin glycosides acylated by a fragment of caffeic acid (λ_max_ 326 nm) [56] (Figure 6). The mass spectrometric data (*m*/*z* 609 [M–H]^–^; MS^2^ 609: 447; MS^3^ 447: 357, 327, 299) indicate that its possible structure is luteolin-*C*-hexoside-*O*-caffeate, especially since a similar compound (isoorientin-2″-O-caffeate) was already isolated from *G. cruciata* [17].

Three luteolin-*C*-hexoside-*O*-di-hexosides (**32**, **36** and **46)** with the same UV pattern gave MS^n^ spectra fragments with *m*/*z* 771, 609, and 447 that are specific for a deprotonated ion and its dehexosylated fragments followed by the further cleavage typical for *C*-glycosylflavones [57]. Possible examples are isoorientin-2″,4′-di-*O*-glucoside isolated from *G. asclepiadea* herb [23] and isoorientin-3″,6″-di-*O*-glucoside from *G. pedicellata* leaves [58]. Two compounds, **65** and **73,** gave a similar MS pattern but the existence of a hypsochromic shift in band II of the UV spectra (λ_max_ 348→328 nm) and increased retention times pointed to acylation of luteolin-*C*-hexoside-*O*-hexoside by a caffeic acid fragment [56]. Despite the relatively rare occurrence of luteolin-*C*-hexoside-*O*-hexoside-*O*-caffeates in plants, there are two known compounds isolated from the gentians, i.e., isoorientin-4′-*O*-glucoside-2″-*O*-caffeate from *G. punctata* leaves [59] and isoorientin-2″-*O*-(4″-*O*-glucosyl)-caffeate from *G. marcailhouana* leaves [60]. Compound **66** from *G. asclepiadea*, *G. paradoxa*, and *G. pneumonanthe* herbs was tentatively identified as luteolin-*C*-hexoside-*O*-hexoside-*O*-*p*-hydroxybenzoate. This was based on its deprotonated ion with *m*/*z* 729 and ions with *m*/*z* 609 and 447 that were caused by the loss of *p*-hydroxybenzoyl and hexosyl fragments, respectively. A flavone glycoside with a similar structure was isolated from *G. asclepiadea* and known as isoorientin-4′-*O*-(2″-*O*-*p*-hydroxybenzoyl)-glucoside [25].

**Figure 7:**
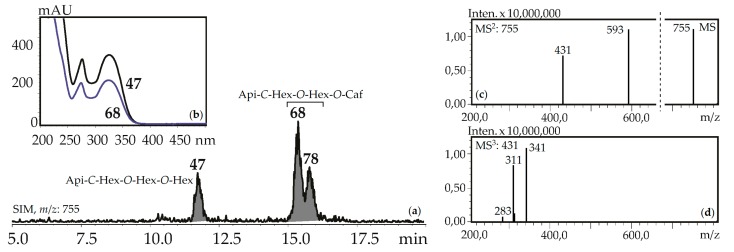
(**a**) HPLC-ESI-MS chromatogram of *G. gelida* herb extract in SIM-mode (*m*/*z* 755). (**b**) UV spectra of compounds **47** and **68**. (**c**) MS and MS^2^ spectra (negative ionization) of compound **68**. Api-*C*-Hex-*O*-Hex-*O*-Hex—apigenin-*C*-hexoside-*O*-hexoside-*O*-hexoside (isovitexin-2″,4″-di-*O*- glucoside), Api-*C*-Hex-*O*-Hex-*O*-Caf—zone of isomeric apigenin-*C*-hexoside-*O*-hexoside-*O*-caffeates.

Fourteen apigenin glycosides were the components of gentian herbs and roots. Apigenin-7-*O*-glucoside (**125**), apigenin-6-*C*-glucoside (isovitexin, **71**) and isovitexin-7-*O*-glucoside (saponarin, **42**) were found in all gentian herb samples, and **42** and **71** were in the roots of *G. asclepiadea*, *G. gelida*, and *G. paradoxa*. Isovitexin is also a frequently detected flavonoid of the gentians [42]. Four *C*,*O*-glycosylflavones were identified by comparing with reference compounds like isovitexin-2″-*O*-glucoside (**55**), isovitexin-4′-*O*-glucoside (**64**), isovitexin-7,2″-di-*O*-glucoside (**37**), and isovitexin-2″,4″-di-*O*-glucoside (**47**). Compounds **57** and **107** are found in some gentians and have spectral data similar to **36** and **97**, respectively, but 16 a.m.u. less, confirming their nature as apigenin-*C*-hexoside-*O*-hexoside-*O*-hexosides and apigenin-*C*-hexoside-*O*-caffeate. Five isomeric compounds (**68**, **78**, **92**, **114**, **118**) with molecular weights 756 were detected in all gentian herbs, and it was concluded that they are acylated *C*,*O*-glycosylflavones or apigenin-*C*-hexoside-*O*-hexoside-*O*-caffeates. Their spectral properties were similar to an apigenin-*C*-hexoside-*O*-hexoside-*O*-hexoside like isovitexin-7,2″-di-*O*-glucoside (**37**) or isovitexin-2″,4″-di-*O*-glucoside (**47**), but the long retention times (15.09–18.09 min) suggested the existence of an additional functional group with high lipophilicity (Figure 7). Because the molecular weight of the loss group was 162 a.m.u., it was identified as caffeoyl. To date, only one compound satisfies these criteria, the isovitexin-7-*O*-(6″-caffeoyl)glucoside isolated from *Bryonia* herbs [61]. The discovery of five isomeric compounds illustrates the necessity for additional study to find other new isomers. 

Five gentians showed the presence of chrysoeriol and its seven glycosides. The known compounds isoscoparin (**85**), isoscoparin-7-*O*-glucoside (**48**), and isoscoparin-2″-*O*-glucoside (**60**) were identified after comparing with reference compounds as well as chrysoeriol-*C*-hexoside-*O*-hexoside (**53**), chrysoeriol-*C*-hexoside-*O*-hexoside-*O*-caffeate (**70**), and two chrysoeriol-*C*-hexoside-*O*-caffeates **104** and **106**. Compounds **48** and **85** were reported once in *G. pneumonanthe* herb [21]. Chrysoeriol and its glycosides are considered to be rare gentian components [42], and its caffeoyl esters are still unknown. Less expected metabolites of the *Gentian* genus are the acacetin derivatives found in our study in *G. cruciata* herb (**87**, **124**) and *G. gelida* herb (**122**). There were acacetin-*C*-hexoside-*O*-hexoside-*O*-caffeate (**87**) and two acacetin-*C*-hexoside-*O*-caffeates (**122**, **124**). Only the isocytisoside-7-*O*-glucoside (acacetin-6-C-glucoside-7-*O*-glucoside) isolated from *G. pyrenaica* herb [62] is a known acacetin glycoside of the gentians. There is not any information about caffeoylated glycosides of acacetin of plant origin. 

#### 2.1.7. Triterpene Glycosides 

**Figure 8:**
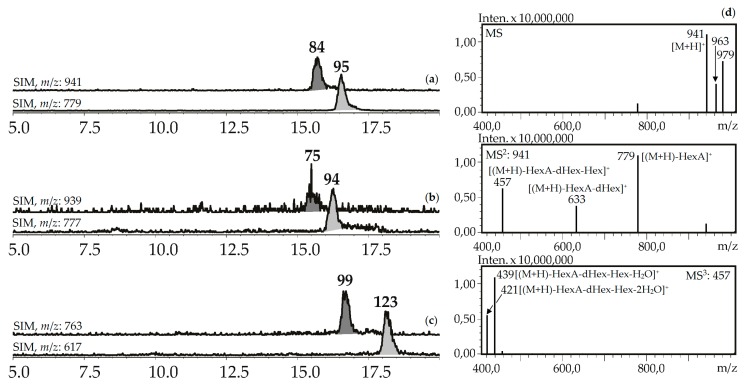
(**a,b,c**) HPLC-ESI-MS chromatograms of *G. asclepiades* herb extract in SIM-mode (positive ionization; *m*/*z* 941, 779 (oleanolic acid glycosides; a), 939, 777 (dehydrooleanolic acid glycosides; b), 763, 617 (desoxyoleanolic acid glycosides; c)). (**d**) MS^n^ spectra (positive ionization) of compound **84**.

Seven compounds (**75**, **84**, **94**, **95**, **99**, **112**, **123**) were determined to be triterpenic glycosides based on their mass spectra in positive ionization mode. Their glycosidic nature was proved by the presence of a series of signals caused by elimination of carbohydrate fragments like hexose (162 a.m.u.), desoxyhexose (146 a.m.u.), and hexuronic acid (176 a.m.u.) (Figure 8). Aglycone fragments in MS^3^ spectra gave the signals of dehydrated ions characteristic of triterpenoids (triterpenes, sterols, ecdysteroids) [63,64]. Two compounds, **84** and **95,** from the herb of *G. asclepiadea*, *G. cruciata* and *G. paradoxa* were determined to be oleanolic acid-*O*-hexuronide-*O*-desoxyhexoside-*O*-hexoside and oleanolic acid-*O*-hexuronide-*O*-desoxyhexoside, respectively. Compounds **75** and **94** have similar mass spectrometric patterns, but the atomic numbers of the basic fragments were 2 a.m.u. less that presumably caused by the absence of two hydrogens in an aglycone fragment. Dehydrooleanolic acid was tentatively concluded to be an aglycone of **75** and **94,** and they may be described as dehydrooleanolic acid-*O*-hexuronide-*O*-desoxyhexoside-*O*-hexoside (**75**) and dehydrooleanolic acid-*O*-hexuronide-*O*-desoxyhexoside (**94**). Compound **123** and two isomers **99** and **112** were desoxyoleanolic acid derivatives coupled with fragments of *O*-hexuronic acid and *O*-hexuronyl-*O*-desoxyhexose, respectively. The known triterpenes of the *Gentiana* genus are mostly derivatives of oleanane, ursane, and dammarane in aglycone state [42]. Triterpene glycosides are rare components of gentians and compounds with the described structural features are still unknown. 

In general, the chemical profiles of the studied gentian species are similar: flavonoids and iridoids accumulate predominately in all types of herbs, and iridoids are the most diverse class of compounds in the roots. Of the 137 compounds detected, 71 compounds (52% of the total) are iridoids and 40 compounds are flavonoids (29% of the total). In total, these account for more than 80% of the total variety of compounds. In gentian herb samples, 44 (*G. pneumonanthe*) to 58 compounds (*G. paradoxa*) were detected and in roots 21 (*G. pneumonanthe*) to 32 compounds (*G. septemfida*). The largest number of compounds known for the species (12) was found in the herb of *G. asclepiadea*, which is considered to be the most studied of the species studied. Most of the compounds mentioned in this study were found in gentian species for the first time (Table 1). Four samples have never been studied before; these include the herb and roots of *G. paradoxa*, and the roots of *G. gelida* and *G. septemfida*.

The common components of all the studied species of both herb and roots were iridoid glycosides, including loganic acid (**18**), loganin (**25**), swertiamarin (**33**), gentiopicroside (**38**), and sweroside (**40**). These compounds are markers for gentian sections of Pneumonanthe and Aptera and the genus *Gentiana*, in general; therefore, their obligate occurrence is not a surprise. Compounds that were found in all herb samples (and rarely in the roots) are glycosylflavones saponarin (**42**), isoorientin-2″-*O*-glycoside (**43**), isoorientin (**61**), isovitexin (**71**), and apigenin-7-*O*-glucoside (**125**). Specific compounds identified mainly in the roots of the studied gentians include some minor iridoid glycosides (**28**, **27**, **126**, **129**, etc.), but these findings are inconclusive and require additional research. 

Is it possible to say that any components are characteristic for a particular type of gentian? The most suitable example is the detection of gentisin (**137**) and gentioside (**109**) in the roots of *G. asclepiadea.* This occasion is really rare, because for gentians of the Pneumonanthe section, the presence of xanthones (except mangiferin) is uncharacteristic. The possible reason for this phenomenon may be the least evolutionary advancement of *G. asclepiadea*, which makes it close to the species of the Coelanthe section, which contains such xanthon-containing species as *G. lutea* and *G. punctata* [42]. 

A notable fact is the single occurrence of some hydroxycinnamates such as caffeoyl-glucose **21** and **45** in *G. pneumonanthe* herb or caffeoyl-glycerins **79** and **90** in *G. septemfida* herb. Commonly, hydroxycinnamates of various structures accumulate in the green parts of plants in the form of esters with quinic acid, shikimic acid, or glucose [65]. For plants of the genus *Gentiana*, such a phenomenon is extremely rare [42]. 

The presence of flavonoids in the form of aglycones was detected only in two species (*G. asclepiadea*, *G. paradoxa*). This is typical for gentian sections of Pneumonanthe and Aptera produced flavonoids in the form of *O-*, *C-*, *C*, *O*-glycosides and most often derivatives of apigenin (or isovitexin) and/or luteolin (or isoorientin) [26,66]. The distribution of xanthone *C*-glycoside mangiferin (**49**) within the genus Gentiana is irregular, and its presence was previously observed only in some types of sections of Pneumonanthe, Frigida, Aptera, and Chondrophyllae [67]. Mangiferin is a useful therapeutic molecule with various bioactivities [68]. New plant sources are needed and *G. paradoxa* herb was shown to be a mangiferin source for the first time. 

### 2.2. HPLC-DAD Quantification of Selected Compounds in Six Caucasian Gentians: Comparison of Herb and Roots

Continuing the metabolomic study of six Caucasian gentians, we determined the quantitative content of selected compounds by high performance liquid chromatography with diode array detection (HPLC-DAD) technique [12]. Six iridoids, nine flavones and mangiferin were chosen as quantitative markers of the herb samples, and roots were analysed using seven iridoids, isoorientin-2″-*O*-glucoside and gentioside (Tables S4, S5, Figure S2). Comparative analysis of quantitative data showed a strong variation of iridoid, flavonoid, and xanthone content in herbs and roots (Table 2). Gentiopicroside was the predominant iridoid glycoside in herb samples of *G. asclepiadea*, *G. cruciata*, and *G. pneumonanthe*, and swertiamarin had maximal content in other herbs of *G. gelida*, *G. paradoxa*, and *G. septemfida*. The loganic acid level in gentian herb was also high and sweroside was a trace compound. Gelidoside was quantified in two herbs, *G. gelida* and *G. septemfida*, and trifloroside content was determined in *G. gelida*, *G. paradoxa* and *G. septemfida*. The main iridoid of roots was gentiopicroside for all samples and its derivative gentiopicroside-6″-*O*-glucoside was quantified only in roots. The earlier information showed a high content of gentiopicroside in *G. asclepiadea*, *G. cruciata*, and *G. pneumonanthe* roots from Hungary (50–60 mg/g) [27], *G. cruciata* herb/roots from East Serbia (10.67/19.57 mg/g; as extract) [22], and *G. pneumonanthe* roots from Serbia (40.02–56.68 mg/g) [18]. In this regard, the herbs and roots of six Caucasian gentians are a good source of gentiopicroside.

The following iridoids were present in much lower amounts: swertiamarin and sweroside in *G. asclepiadea*, *G. cruciata*, and *G. pneumonanthe* roots [27], swertiamarin and sweroside in *G. cruciata* herb/roots extracts [22], swertiamarin, and sweroside in *G. pneumonanthe* herb/roots [18]. By this data, we see a remarkable similarity between known information about iridoid content in gentian parts and the data obtained in the present study.

Flavonoids were quantifiable in all gentian herbs and *G. asclepiadea* roots. The basic flavonoids of herb samples were isoorientin and isoorientin-2″-*O*-glucoside. The content of isovitexin and its *O*-glycosides was less but appropriate for analysis. Isoscoparin was quantifiable in three herb samples such as *G. septemfida*, *G. gelida*, and *G. pneumonanthe*. Only isoorientin-2″-*O*-glucoside was found in *G. asclepiadea* roots. As previously shown, the isoorientin and isovitexin content in *G. pneumonanthe* herb from Serbia was 0.27–2.67 and 0.12–0.88 mg/g, respectively [18]. Isoorientin was mentioned as the dominant flavonoid of some Turkish gentian herbs such as *G. asclepiadea* (1.00–30.72 mg/g), *G. cruciata* (2.41–22.78 mg/g), *G. gelida* (2.64–35.16 mg/g), and *G. septemfida* (1.17–15.19 mg/g) [24]. By contrast to apigenin glycosides, luteolin derivatives were the principal flavonoids with high content in gentian herbs of Caucasus origin and this was also reported in early research of European and Turkish populations of various gentians. 

Xanthone content in gentian herbs was formed by their mangiferin value. The herbs of *G. gelida* and *G. septemfida* were free of mangiferin and other species showed high mangiferin levels. The roots of *G. asclepiadea* accumulate xanthone-*O*-glycoside gentioside at a low level. Mangiferin content was also determined previously in *G. pneumonanthe* herb (0.44–5.81 mg/g) [18] and in *G. asclepiadea* flowers and stem extracts (0.26–1.48 mM) [37]. In view of mangiferin’s importance as a bioactive compound, the herbs of four gentian species could be concluded to be a rich source of this xanthone, especially *G. asclepiadea* herb.

By comparing results of the quantitative profile of herbs and roots of six Caucasian gentians, it can be concluded that the herbs are a good source of iridoids, flavonoids, and xanthones, and the roots can concentrate mainly iridoids. 

### 2.3. Bioactivity of Gentian Extracts as a Function of Phenolic Compounds Content

Antioxidant activity is one of the basic bioactivity properties of plant extracts due to the presence of the various groups of antioxidants. The gentian extracts are no exceptions, and they were previously found to be good antioxidant sources [13,22,39]. In the present work, we studied the antioxidant properties of herb and root extracts of six Caucasian gentians by three methods: 2,2-diphenyl-1-picrylhydrazyl (DPPH) radical scavenging assay, superoxide anion-radical scavenging assay, and lipid peroxidation inhibition assay.

**Figure 9:**
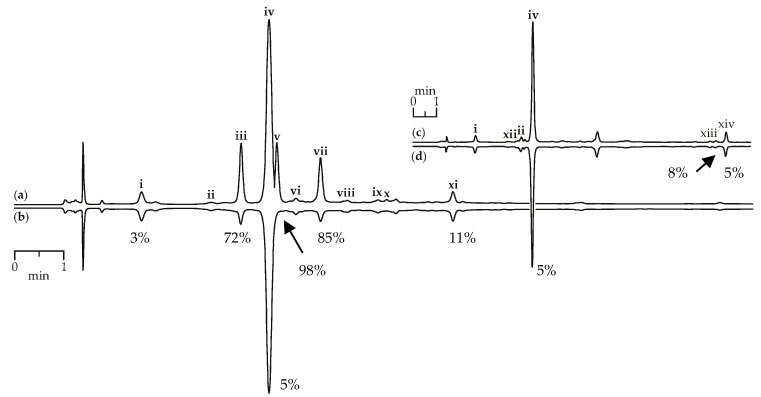
HPLC-DAD chromatograms (210 nm) of *G. asclepiadea* herb extract before (**a**) and after (**b**) pre-chromatographic reaction with DPPH radicals and *G. gelida* roots extract before (**c**) and after (**d**) pre-chromatographic reaction with DPPH radicals. Zone of compounds numbered as follows: i—loganic acid; ii—swertiamarin; iii—isoorientin-2″-*O*-glucoside; iv—gentiopicroside; v—mangiferin; vi—isovitexin-2″-*O*-glucoside; vii—isoorientin; viii—saponarin; ix—isovitexin; x—luteolin-7-*O*-glucoside; xi—apigenin-7-*O*-glucoside; xii—gentiopicroside-6″-*O*-glucoside; xiii—gelidoside (rindoside); xiv—trifloroside. The numbers demonstrate the percentage of peak area reduction after pre-chromatographic reaction with DPPH radicals.

Scavenging activity against DPPH radicals of gentian herb extracts were medium to high and varied from 147.57 mg trolox/g for *G. paradoxa* to 580.71 mg trolox/g for *G. asclepiadea* (Table 3). Extracts of gentian roots were characterized by mostly low activity, with activity values <10 mg trolox/g. Using the HPLC-DAD assay coupled with pre-chromatographic reaction with DPPH radicals, the most active compounds were characterized [69,70]. Figure 9a,b show chromatograms of *G. asclepiadea* herb extract before and after reaction with DPPH radical. The peak areas of the most active scavengers isoorientin-2″-*O*-glucoside (zone iii), mangiferin (zone v), and isoorientin (zone vii) were reduced by 72–98% compared with the initial value, but iridoid glycosides such as loganic acid (zone i) and gentiopicroside (zone iv) gave week peak reduction (3–5%). The chromatograms of root extracts appeared relatively unchanged after reaction with DPPH radicals, demonstrating a low reduction of all peaks (Figure 9c,d). Analysing the scavenging activity of the selected compounds, we found that iridoid glycosides such as loganic acid and gentiopicroside have low activity (<10 mg trolox/g). Additional phenolic groups such as 2,3-dihydroxybenzoyl in iridoid structures of gelidoside (rindoside) and trifloroside can increase the radical scavenging potential, but not much (20.82–25.14 mg trolox/g). Isoorientin (2523.27 mg trolox/g) and mangiferin (3824.20 mg trolox/g) were the most active compounds found in gentian extracts.

The scavenging activity of gentian herb extracts against superoxide anion-radicals was medium to high with values of potential from 52.18 mg quercetin/g for *G. paradoxa* to 235.54 mg quercetin/g for *G. septemfida*, and root extracts had low activity (<10 mg quercetin/g). The lipid peroxidation inhibition values of gentian herb extracts were also high with activities ranging from 63.23–242.08 mg caffeic acid/g. The root extracts had low activity from <10 mg caffeic acid/g to 17.68 mg caffeic acid/g (*G. asclepiadea* roots). The main reason for the high activity of the herb extracts was the high phenolic content (flavonoids, xanthones), which showed the maximum intensity of antioxidant protection. 

**Figure 10:**
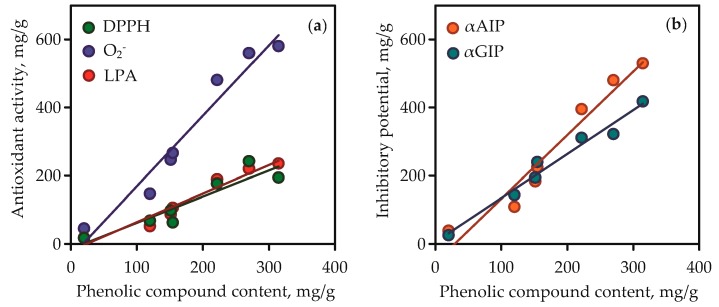
Correlation graphs between phenolic compound content in gentian extracts (mg/g) and their bioactivity. (**a**) DPPH—2,2-diphenyl-1-picrylhydrazyl radical scavenging activity (as mg of trolox per gram of dry extract weight; correlation equation *y* = 2.07·*x* − 38.48, *r* = 0.9752); O_2_^-^—superoxide anion-radical scavenging activity (as mg of quercetin per gram of dry extract weight; correlation equation *y* = 0.84·*x* − 20.21, *r* = 0.9736); LPA—lipid peroxidation inhibition activity (as mg of caffeic acid per g of dry extract weight; correlation equation *y* = 0.76·*x* − 14.74, *r* = 0.9232). (**b**) αAIP—α-amylase inhibitory potential (as mg of acarbose per g of dry extract weight; correlation equation *y* = 1.86·*x* − 54.73, *r* = 0.9739); αGIP—α-glycosidase inhibitory potential (as mg of acarbose per gram of dry extract weight; correlation equation *y* = 1.29·*x* + 5.08, *r* = 0.9849).

Isoorientin and mangiferin demonstrated superoxide anion-radical scavenging activity at 863.15 and 927.07 mg quercetin/g, respectively, and in the lipid peroxidation inhibition assay the parameters of activity for the same compounds were 486.56 and 522.14 mg caffeic acid/g, respectively. It was thus evident that the high content of phenolic compounds such as flavonoids and xanthones in gentian extracts gave them high antioxidant activity. This was clearly confirmed by the results of regression analysis between phenolic compound content in gentian extracts (Table S6) and their antioxidant activity values (Figure 10a). Good linearity was shown for three equations that gave high *r* (correlation coefficient) values from 0.9232 to 0.9752. The fact of the positive direct relationships between phenolic content and antioxidant activity of plant extracts was postulated elsewhere [71], but the compliance of this rule in case of Caucasian gentians was observed for the first time.

The calculated IC_50_ values in the DPPH assay for the Serbian species were 426.67–1000 μg/mL for *G. asclepiadea* root fractions and 181–614 μg/mL for *G. asclepiadea* herb fractions (ascorbic acid as a reference IC_50_ 5.23 μg/mL) [39], for *G. cruciata* herb and roots extracts – 1263 and 2603 μg/mL, respectively (ascorbic acid as a reference IC_50_ 6.05 μg/mL) [22]. The fractions of the *G. septemfida* herb of Turkey origin showed a scavenging effect at a dose of 1000 μg/mL with values of 15.01–80.17% [19]. The superoxide scavenging potential of extracts from the herb and roots of *G. cruciata* (Serbia) was 135 and >1000 μg/mL (gallic acid as a reference IC_50_ 360 μg/mL) [22]. The root fractions of *G. asclepiadea* showed inhibitory activity toward lipid peroxidation with IC_50_ values of 40–183 μg/mL (butylated hydroxytoluene as a reference IC_50_ 1.00 μg/mL) [22], and the extracts of the herb and roots of *G. cruciata* were active with IC_50_ 792 and 894 μg/mL, respectively (butylated hydroxytoluene as a reference IC_50_ 1.00 μg/mL) [22]. All this points out that despite differences in plant origin (Serbia, Turkey, Caucasus), it should be possible to postulate that the high antioxidant activity of gentian herbs makes them a valuable source of antioxidant phytophenolics.

The inhibitory potential of gentian extracts against α-amylase and α-glycosidase as basic digestion enzymes was studied using known spectrophotometric microplate assays [57]. All herb extracts showed high activity in the range 108.85–530.11 mg acarbose/g for α-amylase inhibition and 144.77–418.80 mg acarbose/g for α-glycosidase inhibition. 

The root extracts demonstrated low activity (<10–38.87 mg acarbose/g) for α-amylase inhibition and <10–25.64 mg acarbose/g for α-glycosidase inhibition. The reasons for the varied activity of gentian extracts are in the various chemical profiles and quantitative composition of the extracts. The values of inhibitory potential of pure compounds were low for iridoid glycosides (<10 mg acarbose/g), medium for isovitexin, and high for isoorientin and mangiferin. The latter two compounds were the most active inhibitors, whereas isoorientin was the most potent inhibitor of α-amylase (1242.03 mg acarbose/g in α-amylase inhibition vs. 811.10 mg acarbose/g in α-glycosidase inhibition), and mangiferin was the most potent inhibitor of α-glycosidase (296.14 mg acarbose/g in α-amylase inhibition vs. 1562.84 mg acarbose/g in α-glycosidase inhibition). It is reasonable to expect that the high phenolic content resulted in the high inhibitory activity of gentian extracts against α-amylase and α-glycosidase. Regression equation data showed good linearity for correlation graphs between phenolic compound content in gentian extracts and α-amylase/α-glycosidase inhibitory activity (Figure 10b). In view of all this, there is a need to characterise gentian herbs as potent plant sources of anti-α-amylase and anti-α-glycosidase phenolics.

## 3. Conclusions

The data obtained are in compliance with known facts about high antioxidant and antidiabetic activity of flavones with luteolin skeleton [40,71,72] as well as the xanthone mangiferin [40,68]. Both of these have two phenolic *ortho*-hydroxyl groups. Although Caucasian gentians contain a great number of various phytochemicals, the most abundant active compounds were flavones and mangiferin which resulted in the high efficiency of derived plant extracts as antioxidants and digestive enzymes inhibitors. To our knowledge, this is the first paper combining detailed metabolite profiling by the HPLC-DAD-ESI-QQQ-MS technique and HPLC-DAD quantification of the main compounds with an antioxidant, anti-α-amylase, and anti-α-glycosidase study of the six gentian species (herb, roots) widely distributed in the Caucasus and used as phytopharmaceuticals. 

## 4. Materials and Methods

### 4.1. Plant Materials and Chemicals

The information about samples of gentian herb and roots is listed in Table 4. The species were authenticated by authors Alexey S. Prokopyev (Siberian Botanic Garden, Tomsk State University, Tomsk Russia) and Javanshir I. Isaev (Azerbaijan Medical University, Baku, Azerbaijan). Plant material was dried and powdered before analysis.

The chemicals were purchased from ChemFaces (Wuhan, Hubei, PRC) — chrysoeriol (Cat. No. CFN98785, ≥98%), isoscoparin (Cat. No. CFN90965, ≥98%), morroniside (Cat. No. CFN98161, ≥98%), neomangiferin (Cat. No. CFN98122, ≥98%), sweroside (Cat. No. CFN99455, ≥98%); Extrasynthese (Lyon, France) — amarogentin (Cat. No. 0218S, ≥98%), apigenin-7-*O*-glucoside (Cat. No. 1004 S, ≥99%), orientin (Cat. No. 1054 S, ≥99%); Sigma-Aldrich (St. Louis, MO, USA) — acarbose (Cat. No. A8980, ≥95%), acetonitrile for HPLC (Cat. No 34851, ≥99.9%), α-amylase from *Aspergillus oryzae* (Cat. No. 10065, ~30 U/mg), caffeic acid (Cat. No. C0625, ≥98%), 2,2-diphenyl-1-picrylhydrazyl (Cat. No. D9132), formic acid (Cat. No. F0507, ≥95%), gentiopicroside (Cat. No. SMB00416, ≥98%), α-glucosidase from *Saccharomyces cerevisiae* (Cat. No. G5003, ≥10 units/mg), isomangiferin (Cat. No. PHL83514, ≥98%), isoorientin (Cat. No. 02187, ≥98%), isovitexin (Cat. No. 17804, ≥98%), lithium perchlorate (Cat. No. 431567, ≥99%), loganic acid (Cat. No. SMB00231, ≥95%), loganin (Cat. No. 36483, ≥97%), luteolin-7-*O*-glucoside (Cat. No. 49968, ≥98%), mangiferin (Cat. No. 06279, ≥98%), methanol (Cat. No. 322415, ≥99.8%), perchloric acid 70% (Cat. No. 311421, ≥99%), quercetin (Cat. No. Q4951, ≥95%), saponarin (Cat. No. PHL89784, ≥98%), swertiamarin (Cat. No. 90957, ≥95%), trolox (Cat. No. 238813, ≥97%). Some reference substances were isolated previously as loganic acid-6′-*O*-glucoside, algidiside I, algidiside II, gentiopicroside-6′-*O*-glucoside, sweroside-6′-*O*-glucoside, 6′-*O*-(2,3-dihydroxybenzoyl)-sweroside, gelidoside (rindoside), and trifloroside from *Gentiana algida* [14], swertiamarin-6′-*O*-glucoside from *Gentianella azurea* [73], 1-*O*-caffeoyl-glucose from *Spiraea salicifolia* [74], 6-*O*-caffeoyl-glucose from *Filipendula ulmaria* [75], 2-*O*-caffeoyl-glucaric acid from *Leonurus deminutus* [76], 1,3-di-*O*-caffeoyl-glycerin from *Bupleurum longifolium* [77], gentisin and gentioside from *Anagallidium dichotomum* [78], isoorientin-7-*O*-glucoside, isoorientin-4′-*O*-glucoside, and isovitexin-4′-*O*-glucoside from *Gentiana decumbens* [12], isoorientin-2″-*O*-glucoside from *Silene nutans* [79], isoorientin-6″-*O*-glucoside and isovitexin-2″-*O*-glucoside from *Gastrolychnis tristis* [80], isovitexin-7,2″-di-*O*-glucoside and isovitexin-2″,4″-di-*O*-glucoside from *Melandrium divaricatum* [81], isoscoparin-2″-*O*-glucoside and isoscoparin-7-*O*-glucoside from *Silene aprica* [82].

### 4.2. Total Extract Preparation

To prepare the total extract of gentian herb and roots the powdered sample (100 g) was triple extracted in a conical glass flask (2 L) with 60% methanol (2 L) with stirring and sonication for 60 min at 40 °C with ultrasound power of 100 W and the frequency 35 kHz. The final extracts were filtered through a cellulose filter, combined, evaporated in vacuo until dryness, and stored at 4 °C until further chemical composition analysis and bioactivity assays. The yields of total extracts of gentian herbs and roots listed in Table 4.

### 4.3. High-Performance Liquid Chromatography with Diode Array Detection and Electrospray Ionization Triple Quadrupole Mass Spectrometric Detection (HPLC-DAD-ESI-QQQ-MS) 

Reversed-phase high-performance liquid chromatography with diode array detection and electrospray ionization triple quadrupole mass spectrometric detection (HPLC-DAD-ESI-QQQ-MS) procedure was used for phenolic compounds profiling. Experiments were performed on an LCMS 8050 liquid chromatograph coupled with diode-array-detector and triple-quadrupole electrospray ionization detector (Shimadzu, Columbia, MD, USA), using a GLC Mastro C18 column (150 × 2.1 mm, Ø 3 μm; Shimadzu, Kyoto, Japan), column temperature was 35 °C. Eluent A was 0.5% formic acid in water and eluent B was 0.5% formic acid in acetonitrile. The injection volume was 1 μL, and elution flow was 100 μL/min. Gradient program: 0.0–6.0 min 5–20% B, 6.0–12.0 min 20–40% B, 12.0–16.0 min 40–55% B, 16.0–21.0 min 55–60% B, 21.0–30.0 min 60–100% B, 30.0–35.0 min 100–5% B. The DAD acquisitions were performed in the range of 200–600 nm. MS detection was performed in negative ESI mode using the parameters as follows: temperature levels of ESI interface, desolvation line and heat block were 300 °C, 250 °C and 400 °C, respectively. The flow levels of nebulizing gas (N_2_), heating gas (air) and collision-induced dissociation gas (Ar) were 3 L/min, 10 L/min and 0.3 mL/min, respectively. The MS and MS/MS spectra were both recorded in negative (−3 kV source voltage) and positive mode (+3 kV source voltage) by scanning in the range of *m*/*z* 100–1900 at the collision energy of 10–45 eV. The system was operated under LabSolutions workstation software with the internal LC-MS library.

### 4.4. HPLC-DAD Quantification 

Quantification of iridoid glycosides, flavonoids, and xanthones was realized in mc-HPLC-DAD experiments using microcolumn HPLC apparatus. Experiments were performed on an microcolumn chromatograph Econova MiLiChrom A-02 (Novosibirsk, Russia), using a ProntoSIL-120-5-C18 AQ column (1 × 50 mm, ∅ 1 μm; Metrohm AG; Herisau, Switzerland), column temperature was 30 °C. Eluent A was 0.2 М LiClO_4_ in 0.01 M HClO_4_ and eluent B was 0.01 M HClO_4_ in acetonitrile. The injection volume was 1 μL, and elution flow was 150 μL/min. Gradient program: 0.0–10.0 min 12–35% B, 10.0–15.0 min 35–70% B, 15.0–20.0 min 70–12% B. The chromatograms were recorded at 210 nm. 

To prepare the stock solutions of reference compounds, 18 mg loganic acid, swertiamarin, gelidoside, gentiopicroside, gentiopicroside-6″-*O*-glucoside, sweroside, trifloroside, isovitexin, isovitexin-2″-*O*-glucoside, saponarin, apigenin-7-*O*-glucoside, isoorientin, isoorientin-2″-*O*-glucoside, isoorientin-6″-*O*-glucoside, luteolin-7-*O*-glucoside, isoscoparin, mangiferin, and gentioside were accurately weighed and individually dissolved in methanol in volumetric flask (20 mL). The external standard calibration curve was generated using six data points, covering the concentration 1.75, 14.06, 56.25, 225.00, 450, and 900.00 µg/mL. The calibration curves were created by plotting the peak area vs. the concentration levels. All the analyses were carried out in triplicate and the data were expressed as mean value ± standard deviation (SD). 

For preparation of sample solution, an accurately weighted powdered plant (100 mg) was placed in an Eppendorf tube, 2 mL of 60% methanol was added. Then the sample was extracted twice in an ultrasonic bath for 30 min at 40 °C and centrifuged (3000× *g*, 15 min). Combined supernatants were transferred to volumetric flask (5 mL) and the final volume was reduced to 5 mL. The resultant extract was filtered through a 0.22-μm PTFE syringe filter before injection into the HPLC system for analysis.

### 4.5. Validation Analysis

The linearity of HPLC-DAD quantification method was studied by injecting six concentrations (1.75–900.00 µg/mL) of the 18 reference standards (loganic acid, swertiamarin, gelidoside, gentiopicroside, gentiopicroside-6″-*O*-glucoside, sweroside, trifloroside, isovitexin, isovitexin-2″-*O*-glucoside, saponarin, apigenin-7-*O*-glucoside, isoorientin, isoorientin-2″-*O*-glucoside, isoorientin-6″-*O*-glucoside, luteolin-7-*O*-glucoside, isoscoparin, mangiferin, gentioside). Results from each analysis were averaged and subjected to regression analysis. Limits of detection (LOD) and quantification (LOQ) were determined using the following equations: LOD = (3.3 × *S*_YX_)/*a*; LOQ = (10 × *S*_YX_)/*a*, where *S*_YX_ is a standard deviation of the response (Y intercept) and *a* is a slope of calibration curve. Intra- and inter-day variations, which are presented in terms of percent relative standard deviation (%RSD) of the analyte’s peak area and variability assessed the precision of the HPLC-DAD quantification. For the intra-day variability test, the mixture solution containing 18 reference standards was analysed for five replicates within one day (56.25 µg/mL), while inter-day assay was analysed using the same concentration for intra-day precision on four different days (interval of 1 day). The repeatability test of the sample was performed on 7-fold experiments of the mixture solution contain 18 reference standards (225 µg/mL). The stability test was performed with one sample solution, which was stored at room temperature and analysed at regular intervals (0, 2, 4, 8, 12, 24 and 48 h.). For analysis of recovery data, the appropriate amounts of the powdered sample of 18 reference standards were weighted and spiked with a known amount of reference compound and then analysed five times.

### 4.6. Antioxidant Activity Analysis

The 2,2-diphenyl-1-picrylhydrazyl (DPPH) radical scavenging activity was performed as described earlier [83]. The trolox was used as a positive control (IC_50_ 11.62 ± 0.23 µg/mL), and water was used as a negative control. The IC_50_ value was found as the effective concentration at which DPPH radicals were scavenged by 50%. The value of antioxidant activity (A^DPPH^) against DPPH radicals was found as a ratio of trolox IC_50_ to sample IC_50_ [A^DPPH^ = (IC_50_^Trolox^/ IC_50_^Sample^) × 1000] and expressed as mg of trolox in 1 g of sample. Values are expressed as mean obtained from five independent experiments.

The known assay [84] was used to determine superoxide anion radical scavenging activity. Quercetin was used as a positive control (IC_50_ 21.74 ± 0.42 µg/mL), and water was used as a negative control. The IC_50_ value was found as the effective concentration at which superoxide anion radicals were scavenged by 50%. The value of antioxidant activity (A^O2•^) against superoxide anion radicals was found as a ratio of quercetin IC_50_ to sample IC_50_ [A^O2•^ = (IC_50_^Quercetin^/ IC_50_^Sample^) × 1000] and expressed as mg of quercetin in 1 g of sample. Values are expressed as mean obtained from five independent experiments.

The previously described method [85] was used to investigate the lipid peroxidation inhibition potency. Caffeic acid was used as a positive control (IC_50_ 58.96 ± 1.14 µg/mL), and water was used as a negative control. The IC_50_ value was found as the effective sample concentration gave 50% reduction of optical density of initial solution. The value of lipid peroxidation inhibition (I) was found as a ratio of caffeic acid IC_50_ to sample IC_50_ [I = (IC_50_^Caffeic acid^/ IC_50_^Sample^) × 1000] and expressed as mg of caffeic acid in 1 g of sample. Values are expressed as mean obtained from five independent experiments.

### 4.7. Digestive Enzymes Inhibiting Potential

The α-amylase inhibiting potential and α-glucosidase inhibiting potential were performed using spectrophotometric assays [86,87]. The α-amylase from *Aspergillus oryzae* (3 U/mL) and α-glucosidase from *Saccharomyces cerevisae* (0.5 U/mL) were used as substrates. Acarbose was used as a positive control (IC_50_ 311.14 ± 7.79 µg/mL for α-amylase inhibiting potential; IC_50_ 1282.64 ± 38.46 µg/mL for α-glucosidase inhibiting potential), and water was used as a negative control. The IC_50_ value was found as the effective sample concentration gave 50% inhibition of digestive enzyme. The values of α-amylase/α-glucosidase inhibiting potential (P) was found as a ratio of acarbose IC_50_ to sample IC_50_ [I = (IC_50_^Acarbose^/ IC_50_^Sample^) × 1000] and expressed as mg of acarbose in 1 g of sample. Values are expressed as mean obtained from six independent experiments.

### 4.8. Statistical and Multivariative Analysis

Statistical analyses were performed using a one-way analysis of variance (ANOVA), and the significance of the mean difference was determined by Duncan’s multiple range test. Differences at *p* < 0.05 were considered statistically significant. The results are presented as mean values ± SD (standard deviations) of the three replicates. Advanced Grapher 2.2 (Alentum Software Inc., Ramat-Gan, Israel) was used to perform linear regression analysis and to generate graphs.

## Figures and Tables

**Table 1:** Compounds **1**–**137** found in the herb and roots of six Caucasian *Gentiana* species.

No	*t*_R_, min	Compound	Found in Gentians ^1^											
			Herb						Roots					
			GAS	GCR	GGE	GPA	GPN	GSE	GAS	GCR	GGE	GPA	GPN	GSE
Carbohydrates														
**1**	2.78	*O*-Hexosyl-*O*-hexosyl-hexose ^L^	+	+	+	+	+	+	+	+	+	+	+	+
**2**	2.83	*O*-Hexosyl-hexose ^L^	+	+	+	+	+	+	+	+	+	+	+	+
**4**	3.17	Hexose ^L^	+	+	+	+	+	+						
Iridoid glucosides														
Loganic acid derivatives														
**18**	8.65	Loganic acid ^S^	+	[16]	+	+	+	[11]	+	[15]	+	+	+	+
**15**	7.10	Loganic acid-6′-*O*-Glc ^S^								+				
**58**	13.79	Loganic acid-2′-*O*-DOBA (algidiside I) ^S^		+					+					
**69**	15.22	Loganic acid-6′-*O*-DOBA (algidiside II) ^S^		+				+	+				+	
**77**	15.57	Loganic acid-*O*-DOBA ^L^												+
**59**	13.84	Loganic acid-*O*-DOBA-*O*-Hex ^L^							+					
**62**	14.55	Loganic acid-*O*-DOBA-*O*-Hex ^L^							+					
**82**	15.70	Loganic acid-*O*-Ac_3_-*O*-DOBA-*O*-Hex ^L^					+	+						
**89**	16.06	Loganic acid-*O*-Ac_3_-*O*-DOBA-*O*-Hex ^L^					+		+					
**130**	19.16	Loganic acid-*O*-Ac_3_-*O*-DOBA-*O*-Hex ^L^			+	+		+						
**76**	15.54	Loganic acid-*O*-Caf ^L^									+			
Loganin derivatives														
**25**	9.51	Loganin ^S^	+	+	+	+	+	+	+	+	+	+	+	+
**91**	16.19	Loganin-*O*-DOBA ^L^										+		
**103**	17.00	Loganin-*O*-DOBA ^L^									+	+		
Swertiamarin derivatives														
**33**	10.11	Swertiamarin ^S^	+	[16]	[21]	+	[9]	[11]	[20]	[15]	+	+	[20]	+
**41**	11.28	Swertiamarin isomer ^L^	+			+	+							
**26**	9.58	Swertiamarin-6′-*O*-Glc ^S^								+				
**8**	5.91	Swertiamarin-*O*-Hex		+										
**9**	6.16	Swertiamarin-*O*-Hex		+										
**134**	21.94	Swertiamarin-*O*-Ac_3_-*O*-DOBA ^L^			+									
**93**	16.26	Swertiamarin-*O*-Ac-*O*-DOBA-*O*-Hex ^L^				+							+	
**102**	16.84	Swertiamarin-*O*-Ac-*O*-DOBA-*O*-Hex ^L^										+		+
**108**	17.56	Swertiamarin-*O*-Ac-*O*-DOBA-*O*-Hex ^L^												+
**113**	17.64	Swertiamarin-*O*-Ac_2_-*O*-DOBA-*O*-Hex ^L^			+					+			+	+
**117**	18.08	Swertiamarin-*O*-Ac_2_-*O*-DOBA-*O*-Hex ^L^			+					+			+	+
**131**	19.18	Gelidoside (rindoside) ^S^			+	+		[11]		+	+	+	+	+
**120**	18.10	Swertiamarin-*O*-Ac_3_-*O*-DOBA-*O*-Hex_2_^L^			+						+			+
**128**	18.68	Swertiamarin-*O*-Ac_3_-*O*-DOBA-*O*-Hex_2_^L^			+					+	+			+
Gentiopicroside derivatives														
**38**	11.21	Gentiopicroside ^S^	+	[16]	[21]	+	[10]	[11]	[17]	[15]	+	+	[20]	+
**56**	13.71	Gentiopicroside isomer ^L^	+											
**27**	9.65	Gentiopicroside-6′-*O*-Glc ^S^							[17]	+	+	+		+
**20**	8.69	Gentiopicroside-di-*O*-Hex ^L^							+	+	+			
**74**	15.51	Gentiopicroside-*O*-DOBA ^L^									+			
**81**	15.69	Gentiopicroside-*O*-DOBA ^L^												+
**105**	17.10	Amarogentin ^S^									+			
Sweroside derivatives														
**40**	11.26	Sweroside ^S^	+	[16]	+	+	[9]	[11]	[20]	[15]	+	+	[20]	+
**44**	11.55	Sweroside isomer ^L^	+	+	+	+		+		+	+	+	+	+
**28**	9.67	Sweroside-6′-*O*-Glc ^S^							+	+	+	+		+
**83**	15.71	Sweroside-6′-*O*-DOBA ^S^									+	+		+
**72**	15.43	Sweroside-*O*-DOBA-*O*-Hex ^L^												+
**101**	16.79	Sweroside-*O*-Ac-*O*-DOBA-*O*-Hex ^L^			+	+	+	+		+	+		+	
**110**	17.60	Sweroside-*O*-Ac-*O*-DOBA-*O*-Hex ^L^				+	+	+		+	+	+		
**111**	17.61	Sweroside-*O*-Ac_3_-*O*-DOBA ^L^							+					
**135**	22.42	Sweroside-*O*-Ac_3_-*O*-DOBA ^L^			+	+		+			+			+
**121**	18.11	Sweroside-*O*-Ac_2_-*O*-DOBA-*O*-Hex ^L^			+	+		+				+		+
**127**	18.63	Sweroside-*O*-Ac_2_-*O*-DOBA-*O*-Hex ^L^			+	+		+				+	+	+
**132**	19.67	Trifloroside ^S^			[21]	+		+		+	+	+	+	+
**126**	18.52	Sweroside-*O*-Ac_3_-*O*-DOBA-*O*-Hex_2_^L^									+	+	+	+
**129**	19.14	Sweroside-*O*-Ac_3_-*O*-DOBA-*O*-Hex_2_^L^								+	+	+	+	+
**115**	17.67	Sweroside-*O*-Caf ^L^								+				
Iridoid glucosides with various structures														
88	16.05	Eustomorusside-*O*-Ac_3_-*O*-DOBA-*O*-Hex ^L^			+									
**12**	6.83	Eustoside ^L,T^					+	[11]						
**116**	17.85	Eustomoside-*O*-Ac_3_-*O*-DOBA-*O*-Hex (gentomoside) ^L,T^			+			+						
**133**	19.74	Eustomoside-*O*-Ac_3_-*O*-DOBA-*O*-Hex ^L^				+								
**14**	6.87	Eustomoside ^L,T^		+			+	[11]						
**23**	9.15	Morroniside ^S^						+						
**31**	9.99	Septemfidoside ^L,T^						[11]						
Iridoid glucosides with unknows structures														
**3**	3.05	Iridoid glycoside (MW 408)		+			+	+						
**5**	3.62	Iridoid glycoside (MW 408)		+										
**6**	5.56	Iridoid glycoside (MW 408)		+	+		+	+	+					
**10**	6.39	Iridoid glycoside (MW 408)	+			+	+	+						
**13**	6.85	Iridoid glycoside (MW 408)		+					+					
**17**	7.76	Iridoid glycoside (MW 408)	+			+								
**19**	8.67	Iridoid glycoside (MW 408)	+			+			+					
**96**	16.62	Iridoid glycoside (MW 436)												+
**22**	9.06	Iridoid glycoside (MW 446)		+				+						
**24**	9.25	Iridoid glycoside (MW 446)												+
**7**	5.75	Iridoid glycoside (MW 478)		+										
**86**	15.78	Iridoid glycoside (MW 562)									+			
**100**	16.75	Iridoid glycoside (MW 562)									+	+		
**67**	15.04	Iridoid glycoside (MW 684)										+	+	+
Phenolic acid O-glucosides														
**11**	6.81	2,3-Dihydroxybenzoic acid-*O*-Hex ^L^			+	+	+	+		+	+	+	+	+
**16**	7.45	2,3-Dihydroxybenzoic acid-*O*-Hex ^L^			+	+	+	+		+	+	+	+	+
Hydroxycinnamates														
**21**	8.71	1-*O*-Caffeoyl-glucose ^S^					+							
**45**	11.63	6-*O*-Caffeoyl-glucose ^S^					+							
**51**	12.36	2-*O*-Caffeoyl-glucaric acid ^S^						+						
**79**	15.68	1,3-Di-*O*-caffeoyl-glycerol ^S^						+						
**90**	16.17	1,2-Di-*O*-caffeoyl-glycerol ^L,T^						+						
Xanthones														
**49**	12.22	Mangiferin ^S^	[1]	[14]		+	[8]							
**50**	12.32	Isomangiferin ^S^	+	+		+								
**30**	9.92	Mangiferin isomer ^L^				+								
**39**	11.25	Mangiferin isomer ^L^		+										
**34**	10.14	Mangiferin-6-*O*-Glc ^L,T^	[3]	+		+	+							
**29**	9.76	Mangiferin-7-*O*-Glc (neomangiferin) ^S^	[3]	+		+	+							
**137**	22.85	Gentisin ^S^							[20]					
**109**	17.58	Gentisin-1-*O*-Prim (gentioside) ^S^							+					
Flavonoids														
Luteolin derivatives														
**98**	16.70	Luteolin-7-*O*-Glc ^S^	+		+									
**61**	14.08	Luteolin-6-*C*-Glc (isoorientin) ^S^	[1]	[14]	+	+	[10]	[12]	[18]					
**80**	15.69	Luteolin-8-*C*-Glc (orientin) ^S^	+		+									
**35**	11.07	Isoorientin-7-*O*-Glc ^S^	+											
**43**	11.51	Isoorientin-2″-*O*-Glc ^S^	[1]	+	+	+	+	+	+					
**52**	12.47	Isoorientin-4″-*O*-Glc ^S^	+	+	+	+		+						
**54**	13.34	Isoorientin-6″-*O*-Glc ^S^	+	+	+	+	+							
**63**	14.59	Luteolin-*C*-Hex-*O*-Hex ^L^	+											
**97**	16.63	Isoorientin*-O*-Caf ^L^	+	+	+	+	+	+						
**32**	10.04	Luteolin-*C*-Hex-*O*-Hex_2_^L^			+									
**36**	11.11	Luteolin-*C*-Hex-*O*-Hex_2_^L^	+	+	+									
**46**	11.65	Luteolin-*C*-Hex-*O*-Hex_2_^L^	+		+									
**65**	15.00	Luteolin-*C*-Hex-*O*-Hex-*O*-Caf ^L^	+	+	+	+	+	+						
**73**	15.48	Luteolin-*C*-Hex-*O*-Hex-*O*-Caf ^L^	+	+	+	+		+						
**66**	15.02	Luteolin-*C*-Hex-*O*-Hex-*O*-pHBA ^L^	[4]			+	+							
Apigenin derivatives														
**125**	18.51	Apigenin-7-*O*-Glc ^S^	+	+	+	+	+	+						
**71**	15.41	Isovitexin ^S^	[1]	[14]	+	+	[10]	[13]	[18]		+	+		
**42**	11.46	Isovitexin-7-*O*-Glc (saponarin) ^S^	[3]	+	+	+	[7]	+	+		+	+		
**55**	13.48	Isovitexin-2″-*O*-Glc ^S^	[1]		+	+	+	+						
**64**	14.97	Isovitexin-4′-*O*-Glc ^S^	[1]	[14]										
**37**	11.12	Isovitexin-7,2″-di-*O*-Glc ^S^	+											
**47**	11.78	Isovitexin-2″,4″-di-*O*-Glc ^S^	[2]		+									
**57**	13.77	Apigenin-*C*-Hex-*O*-Hex_2_^L^		+		+								
**107**	17.54	Apigenin-*C*-Hex-*O*-Caf ^L^	+	+	+	+		+						
**68**	15.09	Apigenin-*C*-Hex-*O*-Hex-*O*-Caf ^L^	+	+	+	+								
**78**	15.61	Apigenin-*C*-Hex-*O*-Hex-*O*-Caf ^L^	+	+	+	+	+							
**92**	16.24	Apigenin-*C*-Hex-*O*-Hex-*O*-Caf ^L^		+		+		+						
**114**	17.65	Apigenin-*C*-Hex-*O*-Hex-*O*-Caf ^L^						+						
**118**	18.09	Apigenin-*C*-Hex-*O*-Hex-*O*-Caf ^L^						+						
Chrysoeriol derivatives														
**136**	22.67	Chrysoeriol ^S^	+			+								
**85**	15.77	Isoscoparin ^S^			+	+	[7]	+						
**48**	12.13	Isoscoparin-7-*O*-Glc ^S^			+		[7]	+						
**60**	13.86	Isoscoparin-2″-*O*-Glc ^S^			+		+							
**53**	13.16	Chrysoeriol*-C*-Hex-*O*-Hex ^L^					+							
**70**	15.30	Chrysoeriol*-C*-Hex-*O*-Hex-*O*-Caf ^L^					+							
**104**	17.01	Chrysoeriol*-C*-Hex-*O*-Caf ^L^					+							
**106**	17.34	Chrysoeriol*-C*-Hex-*O*-Caf ^L^			+	+	+	+						
Acacetin derivatives														
**87**	15.89	Acacetin-*C*-Hex-*O*-Hex-*O*-Caf ^L^		+				+						
**122**	18.15	Acacetin-*C*-Hex-*O*-Caf ^L^			+									
**124**	18.20	Acacetin-*C*-Hex-*O*-Caf ^L^		+										
Triterpene glycosides														
**95**	16.56	Oleanolic acid-*O*-HexA-*O*-dHex ^L,T^	+	+		+								
**84**	15.76	Oleanolic acid-*O*-HexA-*O*-dHex-*O*-Hex ^L,T^	+			+								
**94**	16.29	Dehydrooleanolic acid-*O*-HexA-*O*-dHex ^L,T^	+			+	+							
**75**	15.53	Dehydrooleanolic acid-*O*-HexA-*O*-dHex-*O*-Hex ^L,T^	+			+								
**123**	18.17	Desoxyoleanolic acid-*O*-HexA ^L,T^	+			+								
**99**	16.72	Desoxyoleanolic acid-*O*-HexA-*O*-dHex ^L,T^	+			+								
**112**	17.62	Desoxyoleanolic acid-*O*-HexA-*O*-dHex ^L,T^				+								
		Total number of compounds found	49	45	51	58	44	51	25	24	31	27	21	32
		Number of previously found compounds	11	8	3	0	9	10	7	4	0	0	3	0
		Number of compounds found in present study	38	37	48	58	35	41	18	20	31	27	18	32

^1^ Gentian species: GAS—*Gentiana asclepiadea*, GCR—*Gentiana cruciata*, GGE—*Gentiana gelida*, GPA—*Gentiana paradoxa*, GPN—*Gentiana pneumonanthe*, GSE—*Gentiana septemfida*. “+”—presence of compound. Number of the reference describing known data have been retained in square brackets. Abbreviations used: Ac—acetate, Caf—caffeoyl, dHex—desoxyhexose, DOBA—2,3-dihydroxybenzoyl, Glc—glucose, Hex—hexose, HexA—hexuronic acid, MW—molecular weight, pHBA—*p*-hydroxybenzoyl, Prim—primverose (6-*O*-xylosyl-glucose). ^S^ Compound identification was based on comparison with reference standards. ^L^ Compound identification was based on interpretation of UV and MS spectral data and comparison with literature data. ^T^ Tentative identification.

**Table 2:** Content of selected compounds in gentian herbs and roots ^a^, mg/g of dry plant weight (±S.D.).

Compound	GAS	GCR	GGE	GPA	GPN	GSE
Gentian herbs						
Iridoids						
Loganic Acid	11.83 ± 0.21	3.40 ± 0.06	1.97 ± 0.04	3.91 ± 0.08	2.15 ± 0.04	4.07 ± 0.08
Swertiamarin	1.53 ± 0.03	tr.	9.04 ± 0.16	83.06 ± 1.66	tr.	9.47 ± 0.18
Gelidoside	0.00	0.00	7.30 ± 0.14	0.00	0.00	4.66 ± 0.09
Gentiopicroside	91.74 ± 1.85	14.77 ± 0.29	4.41 ± 0.08	79.65 ± 1.59	40.74 ± 0.81	5.08 ± 0.10
Sweroside	tr.	tr.	tr.	tr.	tr.	tr.
Trifloroside	0.00	0.00	2.68 ± 0.05	5.72 ± 0.11	0.00	1.87 ± 0.04
Subtotal Iridoids	105.10	18.17	25.40	172.34	42.89	25.15
Flavonoids						
Isovitexin	0.89 ± 0.02	2.27 ± 0.04	1.05 ± 0.02	0.71 ± 0.02	0.70 ± 0.02	3.59 ± 0.07
Isovitexin-2″-*O*-Glc	1.81 ± 0.04	2.40 ± 0.05	1.39 ± 0.03	3.05 ± 0.06	1.24 ± 0.02	7.03 ± 0.14
Saponarin	1.25 ± 0.03	1.42 ± 0.03	tr.	2.02 ± 0.04	1.09 ± 0.02	0.83 ± 0.02
Apigenin-7-*O*-Glc	2.52 ± 0.07	0.47 ± 0.01	0.67 ± 0.01	0.91 ± 0.02	tr.	tr.
Isoorientin	18.85 ± 0.37	4.78 ± 0.09	33.59 ± 0.67	8.85 ± 0.17	21.26 ± 0.42	17.22 ± 0.34
Isoorientin-2″-*O*-Glc	40.62 ± 0.73	16.05 ± 0.32	15.16 ± 0.30	18.76 ± 0.37	4.53 ± 0.09	19.63 ± 0.39
Isoorientin-6″-*O*-Glc	0.00	0.00	3.22 ± 0.06	0.00	0.00	8.59 ± 0.17
Luteolin-7-*O*-Glc	1.33 ± 0.02	tr.	0.78 ± 0.02	1.79 ± 0.03	1.79 ± 0.03	2.52 ± 0.05
Isoscoparin	0.00	0.00	1.14 ± 0.02	0.00	1.94 ± 0.04	0.40 ± 0.01
Subtotal Flavonoids	67.27	27.39	57.00	36.09	32.55	59.81
Xanthones						
Mangiferin	17.48 ± 0.33	5.75 ± 0.11	0.00	6.45 ± 0.12	3.03 ± 0.06	0.00
Subtotal Xanthones	17.48	5.75	0.00	6.45	3.03	0.00
Total Phenolic Compounds	84.75	33.14	57.00	42.54	35.58	59.81
Total Compounds	189.85	51.31	82.40	214.88	78.47	84.96
Gentian roots						
Iridoids						
Loganic Acid	11.75 ± 0.23	17.31 ± 0.34	6.14 ± 0.12	8.25 ± 0.16	11.28 ± 0.22	8.43 ± 0.16
Swertiamarin	5.87 ± 0.11	2.63 ± 0.05	3.47 ± 0.07	3.53 ± 0.07	3.91 ± 0.08	3.83 ± 0.07
Gelidoside	0.00	0.75 ± 0.02	1.53 ± 0.03	0.42 ± 0.01	0.81 ± 0.02	1.92 ± 0.04
Gentiopicroside	64.71 ± 1.29	57.51 ± 1.15	61.37 ± 1.22	62.76 ± 1.25	56.16 ± 1.14	75.90 ± 1.51
Gentiopicroside-6″-*O*-Glc	1.24 ± 0.02	0.61 ± 0.01	1.21 ± 0.02	0.00	0.84 ± 0.02	5.85 ± 0.11
Sweroside	tr.	3.84 ± 0.07	tr.	3.81 ± 0.07	2.40 ± 0.04	2.15 ± 0.04
Trifloroside	0.00	0.54 ± 0.01	7.07 ± 0.14	3.42 ± 0.06	2.05 ± 0.04	5.60 ± 0.11
Subtotal Iridoids	83.57	83.19	80.79	82.19	77.45	103.68
Flavonoids						
Isoorientin-2″-*O*-Glc	4.43 ± 0.08	0.00	0.00	0.00	0.00	0.00
Subtotal Flavonoids	4.43	0.00	0.00	0.00	0.00	0.00
Xanthones						
Gentioside	0.75 ± 0.02	0.00	0.00	0.00	0.00	0.00
Subtotal Xanthones	0.75	0.00	0.00	0.00	0.00	0.00
Total Phenolic Compounds	5.18	0.00	0.00	0.00	0.00	0.00
Total Compounds	88.75	83.19	80.79	82.19	77.45	103.68

^a^ Gentian species: GAS—*Gentiana asclepiadea*, GCR—*Gentiana cruciata*, GGE—*Gentiana gelida*, GPA—*Gentiana paradoxa*, GPN—*Gentiana pneumonanthe*, GSE—*Gentiana septemfida*. “tr.”—trace content (<LOQ). Abbreviation used: Glc—glucose.

**Table 3:** Parameters of antioxidant activity and α-amylase/α-glycosidase inhibitory potential of gentian extracts and pure compounds ^a,b^.

Extract, Compound	DPPH ^c^	O_2_^- d^	LPA ^e^	αAIP ^f^	αGIP ^f^
*G. Asclepiadea* Herb	580.71 ± 14.52 ^vi^	220.45 ± 6.49 ^xii^	194.56 ± 8.36 ^xviii^	530.11 ± 16.31 ^xxvii^	418.80 ± 12.98 ^xxxv^
*G. Cruciata* Herb	246.34 ± 6.40 ^iv^	86.96 ± 3.14 ^x^	98.54 ± 3.46 ^xvii^	183.48 ± 6.62 ^xxiii^	194.90 ± 5.54 ^xxxii^
*G. Gelida* Herb	481.49 ± 14.43 ^v^	189.14 ± 6.05 ^xi^	176.88 ± 6.37 ^xviii^	395.17 ± 14.02 ^xxv^	311.48 ± 11.78 ^xxxiv^
*G. Paradoxa* Herb	147.57 ± 3.09 ^iii^	52.18 ± 2.03 ^x^	68.17 ± 2.12 ^xvi^	108.85 ± 3.71 ^xxii^	144.77 ± 3.05 ^xxxii^
*G. Pneumonante* Herb	266.96 ± 8.72 ^iv^	106.53 ± 3.62 ^xi^	63.23 ± 4.03 ^xvi^	224.06 ± 8.29 ^xxiv^	240.20 ± 6.91 ^xxxiii^
*G. Septemfida* Herb	560.86 ± 12.18 ^vi^	235.54 ± 7.02 ^xii^	242.08 ± 7.59 ^xix^	480.20 ± 11.94 ^xxvi^	322.02 ± 11.69 ^xxxiv^
*G. Asclepiadea* Roots	45.32 ± 1.17 ^ii^	18.48 ± 0.66 ^ix^	17.68 ± 0.71 ^xv^	38.87 ± 1.51 ^xxi^	25.64 ± 0.97 ^xxx^
*G. Cruciata* Roots	< 10.00	< 10.00	< 10.00	< 10.00	< 10.00
*G. Gelida* Roots	< 10.00	< 10.00	< 10.00	< 10.00	< 10.00
*G. Paradoxa* Roots	< 10.00	< 10.00	< 10.00	< 10.00	< 10.00
*G. Pneumonante* Roots	< 10.00	< 10.00	11.27 ± 0.51 ^xv^	< 10.00	14.23 ± 0.56 ^xxix^
*G. Septemfida* Roots	< 10.00	< 10.00	< 10.00	< 10.00	< 10.00
Loganic Acid	< 10.00	< 10.00	< 10.00	< 10.00	< 10.00
Gentiopicroside	< 10.00	< 10.00	< 10.00	< 10.00	< 10.00
Gelidoside	25.14 ± 0.42 ^i^	< 10.00	< 10.00	< 10.00	< 10.00
Trifloroside	20.82 ± 0.38 ^i^	< 10.00	< 10.00	< 10.00	< 10.00
Isovitexin	425.11 ± 8.50 ^v^	211.63 ± 4.20 ^xii^	96.54 ± 3.73 ^xvii^	108.35 ± 3.79 ^xxii^	52.63 ± 2.09 ^xxxi^
Isoorientin	2523.27 ± 50.46 ^vii^	863.15 ± 17.26 ^xiii^	486.56 ± 19.46 ^xx^	1242.03 ± 44.71 ^xxviii^	811.10 ± 32.44 ^xxxvi^
Mangiferin	3824.20 ± 76.48 ^viii^	927.07 ± 18.54 ^xiv^	522.14 ± 20.85 ^xx^	296.14 ± 10.36 ^xxiv^	1562.84 ± 62.48 ^xxxvii^

^a^ Averages ± standard deviation (S.D.) were obtained from five different experiments. ^b^ Values with different letters (i–xxxvii) indicate statistically significant differences among groups at *p* < 0.05 by one-way ANOVA. ^c^ as mg of trolox per g of dry extract weight; ^d^ as mg of quercetin per g of dry extract weight; ^e^ as mg of caffeic acid per g of dry extract weight; ^f^ as mg of acarbose per g of dry extract weight. DPPH—2,2-diphenyl-1-picrylhydrazyl radical scavenging activity; O_2_^-^—superoxide anion-radical scavenging activity; LPA—lipid peroxidation inhibition activity; αAIP—α-amylase inhibitory potential; αGIP—α-glycosidase inhibitory potential.

**Table 4:** Detailed information of gentiana samples.

Collection Place	Collection Date	Coordinates	Voucher Specimens No	Dry Extract Yield (Herb / Root), % ^a^
*Gentiana asclepiadea* L. (syn. *G. schistocalyx* K.Koch)				
Baş Göynük, Shekinskii District, Azerbaijan	23.VIII.2018	41°11′03.8″N 47°00′29.8″E	AZ/GEN-0818/15-003	28.2 / 25.6
*Gentiana cruciata* L.				
Cek, Gubinskii District, Azerbaijan	18.VI.2018	41°12′25.8″N 48°14′40.6″E	AZ/GEN-0618/11-002	21.4 / 19.6
*Gentiana gelida* M.Bieb.				
Batabat, Shakhbuz District, Azerbaijan	05.VIII.2018	39°32′15.8″N 45°44′10.1″E	AZ/GEN-0818/02-074	25.1 / 23.7
*Gentiana paradoxa* Albov				
Mamdzyshkha, Gagry District, Abkhazia	20.VII.2018	43°18′16.0″N 40°19′37.8″E	AB/GEN-0718/17-109	36.5 / 22.1
*Gentiana pneumonanthe* L.				
Kinghi, Ochamchyrskii District, Abkhazia	15.VII.2018	42°48′46.8″N 41°15′56.2″E	AB/GEN-0718/14-114	23.6 / 20.7
*Gentiana septemfida* Pall. (syn. *G. lagodechiana* (Kusn.) Grossh.)				
Laza, Gusarskii District, Azerbaijan	25.VII.2018	41°03′30.1″N 47°55′36.9″E	AZ/GEN-0718/01-004	24.8 / 26.3

^a^ % of dry plant weight.

## Supplementary Materials

The following are available online at https://www.mdpi.com/2218-1989/9/11/271/s1, Table S1: Ethnopharmacological use of *Gentiana* species by the various Caucasus people, Table S2: Known compounds found in *Gentiana* species mentioned in present study (literature data), Table S3: Retention times and mass spectrometric data of compounds 1–137 found in herb and roots of six Caucasian *Gentiana* species, Table S4: Regression equations, correlation coefficients, standard deviation, limits of detection, limits of quantification and linear ranges for 18 compounds, Table S5: Intra- and inter-day precision, repeatability, stability and recovery for 18 compounds, Table S6: Content of selected phenolic compounds in dry extracts of gentian herbs and roots, Figure S1: High-Performance Liquid Chromatography with Electrospray Ionization Triple Quadrupole Mass Spectrometric Detection chromatogram in base peak chromatogram mode of six *Gentiana* herb and root extracts, Figure S2: Structures of reference compounds, Figure S3: High-Performance Liquid Chromatography with Diode Array Detection chromatograms of gentian herb and roots extracts at 210 nm.
