# Supplementary material for: Caucasian Gentiana Species: Untargeted LC-MS Metabolic Profiling, Antioxidant and Digestive Enzyme Inhibiting Activity of Six Plants
Source: Metabolites. 2019 Nov 7;9(11):271. doi: 10.3390/metabo9110271 (PMC6918269; doi:10.3390/metabo9110271)
Supplement: Supplementary file 1 [file metabolites-09-00271-s001.pdf]

metabolites

Supplementary materials

Caucasian *Gentiana* Species: Untargeted LC-MS Metabolic Profiling, Antioxidant and Digestive Enzyme Inhibiting Activity of Six Plants

Daniil N. Olennikov [1,*], Aydan I. Gadimli [2], Javanshir I. Isaev [2], Nina I. Kashchenko [1], Alexey S. Prokopyev [3], Tatyana N. Kataeva [3], Nadezhda K. Chirikova [4], and Cecile Vennos [5]

1 Laboratory of Medical and Biological Research, Institute of General and Experimental Biology, Siberian Division, Russian Academy of Science, 6 Sakh'yanovoy Street, Ulan-Ude 670047, Russia; ninkk@mail.ru
2 Department of Pharmacognosy, Azerbaijan Medical University, Anvar Gasimzade Street 14, Baku AZ1022, Azerbaijan; aydangadimli25@gmail.com (A.I.G.), isayev.cavanshir@amu.edu.az (J.I.I.)
3 Siberian Botanic Garden, Tomsk State University, Lenin Avenue 34/1, Tomsk 634050, Russia; rareplants@list.ru (A.S.P.), gentianka@mail.ru (T.N.K.)
4 Department of Biochemistry and Biotechnology, North-Eastern Federal University, 58 Belinsky Street, Yakutsk 677027, Russia; hofnung@mail.ru
5 Regulatory and Medical Scientific Affairs, Padma AG, 30 Haldenstrasse, CH-8620 Wetzikon, Switzerland; c.vennos@padma.ch
* Correspondence: olennikovdn@mail.ru; Tel.: +7-9021-600-627 (D.N.O.)

Supplementary content

Table S1. Ethnopharmacological use of *Gentiana* species by the various Caucasus people.

Table S2. Known compounds found in *Gentiana* species mentioned in present study (literature data).

Table S3. Retention times (t_R) and mass spectrometric data of compounds 1–137 found in herb and roots of six Caucasian *Gentiana* species.

Table S4. Regression equations, correlation coefficients (r^2), standard deviation (S_{YX}), limits of detection (LOD), limits of quantification (LOQ) and linear ranges for 18 compounds.

Table S5. Intra- and inter-day precision, repeatability, stability and recovery for 18 compounds.

Table S6. Content of selected phenolic compounds in dry extracts of gentian herbs and roots.

Figure S1. Example of High-Performance Liquid Chromatography with Electrospray Ionization Triple Quadrupole Mass Spectrometric Detection (HPLC-ESI-QQQ-MS) chromatogram in base peak chromatogram mode (BPC mode, negative ionization) and selected ion mode (SIM, negative ionization) of *Gentiana* herb and roots extracts.

Figure S2. Structures of reference compounds.

Figure S3. High-Performance Liquid Chromatography with Diode Array Detection (HPLC-DAD) chromatograms of gentian herb and roots extracts at 210 nm.

Table S1. Ethnopharmacological use of *Gentiana* species by the various Caucasus people.

Gentiana species	Plant part	Description of use [Ref.]
G. asclepiadea	Roots	Diabetes, antipyretic, hepatitis, appetizer (Shekinskii District, Azerbaijan, native population-based data); diarrhea (Adjara) [7]
G. cruciata	Whole plant	Appetizer, antipyretic (Nakhchivan Autonomous Republic, Azerbaijan, native population-based data); anemia (Gubinskii District, Azerbaijan, native population-based data)
	Herb	Stomach pain, malaria, hemorrhoid (Transcaucasia) [8]
G. gelida	Herb	Stomach pain, malaria (Azerbaijan) [7]; diarrhea (Caucasus) [9]
G. paradoxa	Herb	Diabetes, antipyretic, hepatitis, appetizer (Caucasus) [10]
G. pneumonanthe	Whole plant	Diarrhea (Caucasus) [11]
G. septemfida	Whole plant	Appetizer, antipyretic (Nakhichivan Autonomous Republic, Azerbaijan, native population-based data); hepatitis, tuberculosis, bronchitis, pneumonia (Lerikskii District, Azerbaijan, native population-based data); malaria (Azerbaijan) [7,9]
	Herb	Diabetes, antidepressant, antipyretic (Yardymlinskii District, Azerbaijan, native population-based data); stomach pain, wound healing (Astarinskii District, Azerbaijan, native population-based data)
	Roots	Digestive (Yardymlinskii District, Azerbaijan, native population-based data)

Table S2. Known compounds found in *Gentiana* species mentioned in present study (literature data).

No	Compound	Species	Found in [Ref.]	
			Herb	Roots
	Flavonoids			
**1**	Isovitexin	*G. asclepiadea*	[15]	[16]
		G. cruciata	[17]	
		G. pneumonanthe	[18]	
		G. septemfida	[19]	
**2**	Isovitexin-7-*O*-glucoside (saponarin)	*G. asclepiadea*	[20]	
		G. pneumonanthe	[21]	
**3**	Isovitexin-4′-*O*-glucoside	*G. asclepiadea*	[15]	
		G. cruciata	[17]	
**4**	Isovitexin-7,4′-di-*O*-glucoside	*G. cruciata*	[22]	
**5**	Isovitexin-2′′-*O*-glucoside	*G. asclepiadea*	[15]	
**6**	Isovitexin-2′′,4′-di-*O*-glucoside	*G. asclepiadea*	[23]	
**7**	Vitexin	*G. cruciata*	[22]	[22]
**8**	Isoorientin	*G. asclepiadea*	[15]	[16]
		G. cruciata	[17]	
		G. pneumonanthe	[18]	
		G. septemfida	[19,24]	
**9**	Isoorientin 2′′-*O*-*p*-hydroxybenzoyl ester	*G. asclepiadea*	[25]	
**10**	Isoorientin 2′′-*O*-caffeoyl ester	*G. cruciata*	[17]	
**11**	Isoorientin-4′-*O*-glucoside	*G. asclepiadea*	[15]	
		G. cruciata	[17]	
**12**	Isoorientin-4′-*O*-(2′′-*O*-*p*-hydroxybenzoyl)-glucoside	*G. asclepiadea*	[25]	
**13**	Isoorientin-2′′-*O*-glucoside	*G. asclepiadea*	[15]	
**14**	Isoorientin-2′′,4′-di-*O*-glucoside	*G. asclepiadea*	[23]	
**15**	Orientin	*G. cruciata*	[22]	
**16**	Isoscoparin	*G. pneumonanthe*	[21]	
**17**	Isoscoparin-7-*O*-glucoside	*G. pneumonanthe*	[21]	
	Xanthones			
**18**	Mangiferin	*G. asclepiadea*	[15]	[16]
		G. cruciata	[17]	
		G. pneumonanthe	[18,26]	
**19**	Mangiferin-6-*O*-glucoside	*G. asclepiadea*	[20]	
**20**	Mangiferin-7-*O*-glucoside	*G. asclepiadea*	[20]	
**21**	Gentisin	*G. asclepiadea*		[27]
		G. pneumonanthe		[27]
**22**	Isogentisin	*G. asclepiadea*		[16]
	Iridoids			
**23**	Eustoside	*G. septemfida*	[28]	
**24**	Eustomoside	*G. gelida*	[29]	
		G. septemfida	[28]	
**25**	Eustomorusside	*G. gelida*	[29]	
		G. septemfida	[28]	
**26**	Gelidoside	*G. gelida*	[29]	
		G. septemfida	[28]	
**27**	Gentiopicroside	*G. asclepiadea*		[27,30]
		G. cruciata	[22]	[22,27,31]
		G. gelida	[29]	
		G. pneumonanthe	[18]	[27]
		G. septemfida	[19,28]	
**28**	Gentiopicroside-6′-*O*-glucoside	*G. asclepiadea*		[30]
**29**	Loganic acid	*G. cruciata*	[22]	[22,31]
		G. septemfida	[28]	
**30**	Septemfidoside	*G. septemfida*	[28]	
**31**	Swertiamarin	*G. asclepiadea*		[27]
		G. cruciata	[22]	[22,27,31]
		G. gelida	[29]	
		G. pneumonanthe	[18,32]	[27]
		G. septemfida	[19,28]	

Table S2. Continuation

No	Compound	Species	Found in [Ref.]	
			Herb	Roots
**32**	Sweroside	*G. asclepiadea*		[27]
		G. cruciata	[22]	[22,27,31]
		G. pneumonanthe	[18,32]	[27]
		G. septemfida	[28]	
**33**	Gentomoside	*G. gelida*	[29]	
**34**	Trifloroside	*G. gelida*	[29]	
**35**	Amarogentin	*G. asclepiadea*		[27]
		G. pneumonanthe		[27]
	Monoterpenes			
**36**	Pneumonanthoside	*G. pneumonanthe*	[32]	
	Triterpenes			
**37**	Sitosterol	*G. asclepiadea*		[16]
**38**	Ursolic acid	*G. asclepiadea*		[16]
	Various			
**39**	3,4-Dihydro-1H,6H,8H-naphtho[1,2-c:4,5-c',d']dipyrano-1,8-dione	*G. asclepiadea*		[33]

Table S3. Retention times (t_R) and mass spectrometric data of compounds 1–137 found in herb and roots of six Caucasian *Gentiana* species.

No	t_R, min	Compound [Ref.]	Negative ionization			Positive ionization		
			[M–H]⁻	Adduct ions [a]	MS/MS	[M+H]⁺	Adduct ions [a]	MS/MS
1	2.78	*O*-Hexosyl-*O*-hexosyl-hexose [L] [41]	503	549 [A1]		505	527 [A2]<br>543 [A3]	**[505]**: 343<br>**[343]**: 181
2	2.83	*O*-Hexosyl-hexose [L] [41]	341	387 [A1]		343	365 [A2]<br>381 [A3]	**[343]**: 181
3	3.05	Iridoid glycoside (MW 408) [L] [28]	407	453 [A1]	**[407]**: 245	409	431 [A2]<br>447 [A3]	
4	3.17	Hexose [L] [41]	179	225 [A1]		181	203 [A2]<br>219 [A3]	
5	3.62	Iridoid glycoside (MW 408) [L] [28]	407	453 [A1]	**[407]**: 245	409	431 [A2]<br>447 [A3]	
6	5.56	Iridoid glycoside (MW 408) [L] [28]	407	453 [A1]	**[407]**: 245	409	431 [A2]<br>447 [A3]	
7	5.75	Iridoid glycoside (MW 478) [L]	477	523 [A1]	**[477]**: 315	479	501 [A2]<br>518 [A3]	
8	5.91	Swertiamarin-*O*-hexoside [L] [12]	535	581 [A1]	**[535]**: 373, 211	537	559 [A2]<br>575 [A3]	**[537]**: 375
9	6.16	Swertiamarin-*O*-hexoside [L] [12]	535	581 [A1]	**[535]**: 373, 211	537	559 [A2],<br>575 [A3]	**[537]**: 375
10	6.39	Iridoid glycoside (MW 408) [L] [28]	407	453 [A1]	**[407]**: 245	409	431 [A2],<br>447 [A3]	
11	6.81	2,3-Dihydroxybenzoic acid-*O*-hexoside [L] [55]	315		**[315]**: 153	317		
12	6.83	Eustoside [L,T] [28]	425	471 [A1]	**[425]**: 263	427	449 [A2]<br>465 [A3]	
13	6.85	Iridoid glycoside (MW 408) [L] [28]	407	453 [A1]	**[407]**: 245	409	431 [A2]<br>447 [A3]	
14	6.87	Eustomoside [L,T] [43]	389	435 [A1]	**[389]**: 227	391	413 [A2]<br>429 [A3]	
15	7.10	Loganic acid-6′-*O*-glucoside [S] [14]	537	583 [A1]	**[537]**: 375, 213	539	561 [A2]<br>577 [A3]	**[539]**: 377
16	7.45	2,3-Dihydroxybenzoic acid-*O*-hexoside [L] [55]	315		**[315]**: 153	317		
17	7.76	Iridoid glycoside (MW 408) [L] [28]	407	453 [A1]	**[407]**: 245	409	431 [A2]<br>447 [A3]	
18	8.65	Loganic acid [S] [14]	375	421 [A1]	**[375]**: 213	377	399 [A2]<br>415 [A3]	
19	8.67	Iridoid glycoside (MW 408) [L] [28]	407	453 [A1]		409	431 [A2]<br>447 [A3]	
20	8.69	Gentiopicroside-di-*O*-hexoside [L] [44]	679	725 [A1]	**[679]**: 517, 355<br>**[355]**: 193	681	703 [A2]<br>719 [A3]	**[681]**: 519, 357
21	8.71	1-*O*-Caffeoyl-glucose [S] [42]	341		**[341]**: 179	343		**[343]**: 181
22	9.06	Iridoid glycoside (MW 446) [L] [44]	445	491 [A1]	**[445]**: 283	447	469 [A2]<br>485 [A3]	
23	9.15	Morroniside [S] [44]	405	451 [A1]	**[405]**: 243	407	429 [A2]<br>445 [A3]	

Table S3. Continuation

No	t_R, min	Compound [Ref.]	Negative ionization			Positive ionization		
			**[M–H]$^-$**	**Adduct ions ^{a}**	**MS/MS**	**[M+H]$^+$**	**Adduct ions ^{a}**	**MS/MS**
24	9.25	Iridoid glycoside (MW 446) ^{L} [44]	445	491 ^{A1}	[**445**]: 283	447	469 ^{A2}<br>485 ^{A3}	
25	9.51	Loganin ^{S} [12]	389	435 ^{A1}	[**389**]: 227	391	413 ^{A2}<br>429 ^{A3}	
26	9.58	Swertiamarin-6′-*O*-glucoside ^{S} [12]	535	581 ^{A1}	[**535**]: 373<br>[**373**]: 211	537	559 ^{A2}<br>575 ^{A3}	[**537**]: 375
27	9.65	Gentiopicroside-6′-*O*-glucoside ^{S} [44]	517	563 ^{A1}	[**517**]: 355<br>[**355**]: 193	519	541 ^{A2}<br>557 ^{A3}	[**519**]: 357
28	9.67	Sweroside-6′-*O*-glucoside ^{S} [44]	519	565 ^{A1}	[**519**]: 357<br>[**357**]: 195	521	543 ^{A2}<br>559 ^{A3}	[**521**]: 359
29	9.76	Mangiferin-7-*O*-glucoside (neomangiferin) ^{S} [20]	583	629 ^{A1}	[**583**]: 421	585		[**585**]: 423
30	9.92	Mangiferin isomer ^{L} [15,17,26]	421	467 ^{A1}		423		
31	9.99	Septemfidoside ^{T,L} [28]	765	811 ^{A1}		767	789 ^{A2}<br>805 ^{A3}	
32	10.04	Luteolin-*C*-hexoside-*O*-hexoside-*O*-hexoside ^{L} [23,57,58]	771		[**771**]: 609, 447<br>[**609**]: 447<br>[**447**] 357, 327, 299	773		[**773**]: 611<br>[**611**]: 449
33	10.11	Swertiamarin ^{S} [12]	373	419 ^{A1}	[**373**]: 211	375	397 ^{A2}<br>415 ^{A3}	
34	10.14	Mangiferin-6-*O*-glucoside ^{T} [20]	583	629 ^{A1}	[**583**]: 421	585		[**585**]: 423
35	11.07	Isoorientin-7-*O*-glucoside ^{S} [14–17]	609		[**609**]: 447<br>[**447**] 357, 327, 329, 299	611		[**611**]: 449
36	11.11	Isoorientin-2′′,4′′-di-*O*-glucoside ^{S} [23,57,58]	771		[**771**]: 609, 447<br>[**609**]: 447<br>[**447**] 357, 327, 329, 299	773		[**773**]: 611<br>[**611**]: 449
37	11.12	Isovitexin-7,2′′-di-*O*-glucoside ^{S} [14–17]	755		[**755**] 593<br>[**593**] 431<br>[**431**] 341, 311, 313, 283	757		[**757**] 595<br>[**595**] 433
38	11.21	Gentiopicroside ^{S} [12]	355	401 ^{A1}	[**355**]: 193	357	379 ^{A2}<br>395 ^{A3}	
39	11.25	Mangiferin isomer ^{L} [15,17,26]	421	467 ^{A1}		423		
40	11.26	Sweroside ^{S} [12]	357	403 ^{A1}	[**357**]: 195	359	381 ^{A2}<br>397 ^{A3}	
41	11.28	Swertiamarin isomer ^{L} [12]	373	419 ^{A1}	[**373**]: 211	375	397 ^{A2}<br>415 ^{A3}	
42	11.46	Isovitexin-7-*O*-glucoside (saponarin) ^{S} [57]	593		[**593**] 431<br>[**431**] 341, 311, 313, 283	595		[**595**] 433
43	11.51	Isoorientin-2′′-*O*-glucoside ^{S} [14–17]	609		[**609**]: 447<br>[**447**] 357, 327, 329, 299	611		[**611**]: 449

Table S3. Continuation

No	tR, min	Compound [Ref.]	Negative ionization			Positive ionization		
			[M–H]⁻	Adduct ions [a]	MS/MS	[M+H]⁺	Adduct ions [a]	MS/MS
44	11.55	Sweroside isomer [L] [12]	357	403 [A1]	[**357**]: 195	359	381 [A2]<br>397 [A3]	
45	11.63	6-*O*-Caffeoyl-glucose [S] [42]	341		[**341**]: 179	343		[**343**]: 181
46	11.65	Luteolin-*C*-hexoside-*O*-hexoside-*O*-hexoside [L] [23,57,58]	771		[**771**]: 609, 447<br>[**609**]: 447<br>[**447**] 357, 327, 299	773		[**773**]: 611<br>[**611**]: 449
47	11.78	Isovitexin-2′′,4′′-di-*O*-glucoside [S] [14–17]	755		[**755**] 593<br>[**593**] 431<br>[**431**] 341, 311, 313, 283	757		[**757**] 595<br>[**595**] 433
48	12.13	Isoscoparin-7-*O*-glucoside [S] [21]	623		[**623**] 461<br>[**461**] 371, 341, 343, 313	625		[**625**] 463
49	12.22	Mangiferin [S] [15,17,26]	421	467 [A1]		423		
50	12.32	Isomangiferin [S] [15,17,26]	421	467 [A1]		423		
51	12.36	2-*O*-Caffeoyl-glucaric acid [S] [76]	371	417 [A1]	[**371**]: 209	373		
52	12.47	Isoorientin-4′′-*O*-glucoside [S] [14–17]	609		[**609**]: 447<br>[**447**] 357, 327, 329, 299	611		[**611**]: 449
53	13.16	Chrysoeriol-*C*-hexoside-*O*-hexoside [L] [21,42,57]	623		[**623**] 461<br>[**461**] 371, 341, 343, 313	625		[**625**] 463
54	13.34	Isoorientin-6′′-*O*-glucoside [S] [14–17]	609		[**609**]: 447<br>[**447**] 357, 327, 329, 299	611		[**611**]: 449
55	13.48	Isovitexin-2′′-*O*-glucoside [S] [14–17]	593		[**593**] 431<br>[**431**] 341, 311, 313, 283	595		[**595**] 433
56	13.71	Gentiopicroside isomer [L] [12]	355	401 [A1]	[**355**]: 193	357	379 [A2]<br>395 [A3]	
57	13.77	Apigenin-*C*-hexoside-*O*-hexoside-*O*-hexoside [L] [23,57,58]	755		[**755**] 593<br>[**593**] 431<br>[**431**] 341, 311, 313, 283	757		[**757**] 595<br>[**595**] 433
58	13.79	Algidiside I [S] [14]	511	557 [A1]	[**511**] 375<br>[**375**] 213	513	535 [A2]<br>551 [A3]	[**513**] 377
59	13.84	Loganic acid-*O*-DOBA-*O*-hexoside [L] [47,48]	673	719	[**673**] 511<br>[**511**] 375<br>[**375**] 213	675	697 [A2]<br>713 [A3]	[**675**] 513<br>[**513**] 377
60	13.86	Isoscoparin-2′′-*O*-glucoside [S] [21]	623		[**623**] 461<br>[**461**] 371, 341, 343, 313	625		[**625**] 463
61	14.08	Isoorientin [S] [57]	447		[**447**] 357, 327, 329, 299	449		
62	14.55	Loganic acid-*O*-DOBA-*O*-hexoside [L] [47,48]	673	719	[**673**] 511<br>[**511**] 375<br>[**375**] 213	675	697 [A2]<br>713 [A3]	[**675**] 513<br>[**513**] 377

Table S3. Continuation

No	tR, min	Compound [Ref.]	Negative ionization			Positive ionization		
			[M–H]⁻	Adduct ions [a]	MS/MS	[M+H]⁺	Adduct ions [a]	MS/MS
63	14.59	Luteolin-*C*-hexoside-*O*-hexoside [L] [14–17]	609		[**609**]: 447 [**447**] 357, 327, 329, 299	611		[**611**]: 449
64	14.97	Isovitexin-4′-*O*-glucoside [S] [14–17]	593		[**593**] 431 [**431**] 341, 311, 313, 283	595		[**595**] 433
65	15.00	Luteolin-*C*-hexoside-*O*-hexoside-*O*-Caf [L] [56,59,60]	771		[**771**]: 609, 447 [**609**]: 447 [**447**] 357, 327, 299	773		[**773**]: 611 [**611**]: 449
66	15.02	Luteolin-*C*-hexoside-*O*-hexoside-*O*-pHBA [L] [25]	729		[**729**]: 609, 447 [**447**] 357, 327, 299	731		[**731**]: 611 [**611**]: 449
67	15.04	Iridoid glycoside (MW 684) [L]	683	729 [A1]		685	707 [A2] 723 [A3]	
68	15.09	Apigenin-*C*-hexoside-*O*-hexoside-*O*-Caf [L] [23,57,58,61]	755		[**755**] 593 [**593**] 431 [**431**] 341, 311, 313, 283	757		[**757**] 595 [**595**] 433
69	15.22	Algidiside II [S] [14]	511	557 [A1]	[**511**] 375 [**375**] 213	513	535 [A2] 551 [A3]	[**513**] 377
70	15.30	Chrysoeriol-*C*-hexoside-*O*-hexoside-*O*-Caf [L] [21,42,57]	785		[**785**] 623 [**623**] 461 [**461**] 371, 341, 343, 313	787		[**787**] 625, 463
71	15.41	Isovitexin [S] [57]	431		[**431**] 341, 311, 313, 283	433		
72	15.43	Sweroside-*O*-DOBA-*O*-hexoside [L] [47,48]	655	701 [A1]	[**655**] 493 [**493**] 357 [**357**] 195	657	679 [A2] 695 [A3]	[**657**] 495, 359
73	15.48	Luteolin-*C*-hexoside-*O*-hexoside-*O*-Caf [L] [56,59,60]	771		[**771**]: 609, 447 [**609**]: 447 [**447**] 357, 327, 299	773		[**773**]: 611 [**611**]: 449
74	15.51	Gentiopicroside-*O*-DOBA [L] [44]	491	537 [A1]	[**491**]: 355 [**355**]: 193	493	515 [A2] 531 [A3]	[**493**]: 357
75	15.53	Dehydrooleanolic acid-*O*-hexuronide-*O*-desoxyhexoside-*O*-hexoside [L] [63,64]	937	983 [A1]	[**937**]: 453	939	961 [A2] 977 [A3]	[**939**]: 777, 631, 455 [**455**]: 437, 419
76	15.54	Loganic acid-*O*-Caf [L] [53,54]	537	583 [A1]	[**537**]: 375, 213	539	561 [A2] 577 [A3]	[**539**]: 377
77	15.57	Loganic acid-*O*-DOBA [L] [14]	511	557 [A1]	[**511**] 375 [**375**] 213	513	535 [A2] 551 [A3]	[**513**] 377

Table S3. Continuation

No	t_R, min	Compound [Ref.]	Negative ionization			Positive ionization		
			[M–H]⁻	Adduct ions [a]	MS/MS	[M+H]⁺	Adduct ions [a]	MS/MS
78	15.61	Apigenin-*C*-hexoside-*O*-hexoside-*O*-Caf [L] [23,57,58,61]	755		[**755**] 593<br>[**593**] 431<br>[**431**] 341, 311, 313, 283	757		[**757**] 595<br>[**595**] 433
79	15.68	1,3-Di-*O*-caffeoyl-glycerol [S] [77]	415	461 [A1]	[**415**] 253	417		
80	15.69	Orientin [S] [57]	447		[**447**] 357, 327, 329, 299	449		
81	15.69	Gentiopicroside-*O*-DOBA [L] [44]	491	537 [A1]	[**491**]: 355<br>[**355**]: 193	493	515 [A2]<br>531 [A3]	[**493**]: 357
82	15.70	Loganic acid-*O*-Ac3-*O*-DOBA-*O*-hexoside [L] [12,29,49]	799	845 [A1]	[**799**]: 637, 501, 375<br>[**375**]: 213	801	823 [A2]<br>839 [A3]	[**801**]: 377
83	15.71	Sweroside-6´-*O*-DOBA [S] [44]	493	539 [A1]	[**493**] 357<br>[**357**] 195	495	517 [A2]<br>533 [A3]	[**495**]: 359
84	15.76	Oleanolic acid-*O*-hexuronide-*O*-desoxyhexoside-*O*-hexoside [L] [63,64]	939	985 [A1]	[**939**]: 455	941	963 [A2]<br>979 [A3]	[**941**]: 779, 633, 457<br>[**457**]: 439, 421
85	15.77	Isoscoparin [S] [21]	461		[**461**] 371, 341, 343, 313	463		
86	15.78	Iridoid glycoside (MW 562) [L] [28]	561	607 [A1]		563	585 [A2]<br>601 [A3]	
87	15.89	Acacetin-*C*-hexoside-*O*-hexoside-*O*-Caf [L] [56,87]	769		[**769**] 607<br>[**607**] 445<br>[**445**] 355, 325, 327, 297	771		[**771**] 609<br>[**609**] 447
88	16.05	Eustomorusside-*O*-Ac3-*O*-DOBA-*O*-hexoside [L] [28,29,46]	831	877 [A1]	[**831**] 669, 533, 407<br>[**407**] 245	833	855 [A2]<br>871 [A3]	[**833**] 409
89	16.06	Loganic acid-*O*-Ac3-*O*-DOBA-*O*-hexoside [L] [12,29,49]	799	845 [A1]	[**799**]: 637, 501, 375<br>[**375**]: 213	801	823 [A2]<br>839 [A3]	[**801**]: 377
90	16.17	1,2-Di-*O*-caffeoyl-glycerol [L,T] [77]	415	461 [A1]	[**415**] 253	417		
91	16.19	Loganin-*O*-DOBA [L] [44]	525	571 [A1]	[**525**] 389<br>[**389**] 227	527	549 [A2]<br>565 [A3]	[**527**]: 391
92	16.24	Apigenin-*C*-hexoside-*O*-hexoside-*O*-Caf [L] [23,57,58,61]	755		[**755**] 593<br>[**593**] 431<br>[**431**] 341, 311, 313, 283	757		[**757**] 595<br>[**595**] 433
93	16.26	Swertiamarin-*O*-Ac-*O*-DOBA-*O*-hexoside [L] [29,49]	713	759 [A1]	[**713**] 551, 415, 373<br>[**373**] 211	715	737 [A2]<br>753 [A3]	[**715**] 375
94	16.29	Dehydrooleanolic acid-*O*-hexuronide-*O*-desoxyhexoside [L] [63,64]	775	821 [A1]	[**775**]: 453	777	799 [A2]<br>815 [A3]	[**777**]: 631, 455<br>[**455**]: 437, 419
95	16.56	Oleanolic acid-*O*-hexuronide-*O*-desoxyhexoside [L] [63,64]	777	823 [A1]	[**777**]: 455	779	801 [A2]<br>817 [A3]	[**779**]: 633, 457<br>[**457**]: 439, 421
96	16.62	Iridoid glycoside (MW 562) [L] [28]	561	607 [A1]		563	585 [A2]<br>601 [A3]	

Table S3. Continuation

No	t_R, min	Compound [Ref.]	Negative ionization			Positive ionization		
			$[M–H]^-$	Adduct ions ^{a}	MS/MS	$[M+H]^+$	Adduct ions ^{a}	MS/MS
97	16.63	Isoorientin-*O*-Caf ^{L} [17,56]	609		[**609**]: 447 [**447**] 357, 327, 329, 299	611		[**611**]: 449
98	16.70	Luteolin-7-*O*-glucoside ^{S} [57]	447		[**447**] 285	449		[**449**] 287
99	16.72	Desoxyoleanolic acid-*O*-hexuronide-*O*-desoxyhexoside ^{L} [63,64]	761	807 ^{A1}	[**761**]: 439	763	785 ^{A2} 801 ^{A3}	[**785**]: 617, 441 [**441**]: 423
100	16.75	Iridoid glycoside (MW 562) ^{L} [28]	561	607 ^{A1}		563	585 ^{A2} 601 ^{A3}	
101	16.79	Sweroside-*O*-Ac-*O*-DOBA-*O*-hexoside ^{L}	697	743 ^{A1}	[**697**]: 535, 399, 357 [**357**]: 195	699	721 ^{A2} 737 ^{A3}	[**699**]: 537, 359
102	16.84	Swertiamarin-*O*-Ac-*O*-DOBA-*O*-hexoside ^{L} [29,49]	713	759 ^{A1}	[**713**] 551, 415, 373 [**373**] 211	715	737 ^{A2} 753 ^{A3}	[**715**] 375
103	17.00	Loganin-*O*-DOBA ^{L} [44]	525	571 ^{A1}	[**525**] 389 [**389**] 227	527	549 ^{A2} 565 ^{A3}	[**527**]: 391
104	17.01	Isoscoparin-*O*-Caf ^{L} [21,42,57]	623		[**623**] 461 [**461**] 371, 341, 343, 313	625		[**625**]: 463
105	17.10	Amarogentin ^{S} [27]	585	631 ^{A1}		587	609 ^{A2} 625 ^{A3}	
106	17.34	Isoscoparin-*O*-Caf ^{L} [21,42,57]	623		[**623**] 461 [**461**] 371, 341, 343, 313	625		[**625**]: 463
107	17.54	Isovitexin-*O*-Caf ^{L} [23,57,58]	593		[**593**] 431 [**431**] 341, 311, 313, 283	595		[**595**]: 433
108	17.56	Swertiamarin-*O*-Ac-*O*-DOBA-*O*-hexoside ^{L} [29,49]	713	759 ^{A1}	[**713**] 551, 415, 373 [**373**] 211	715	737 ^{A2} 753 ^{A3}	[**715**] 375
109	17.58	Gentisin-1-*O*-primveroside (gentioside) ^{S} [27]	551	597 ^{A1}	[**551**] 419, 257	553	575 ^{A2}	[**553**] 259
110	17.60	Sweroside-*O*-Ac-*O*-DOBA-*O*-hexoside ^{L} [49–51]	697	743 ^{A1}	[**697**]: 535, 399, 357 [**357**]: 195	699	721 ^{A2} 737 ^{A3}	[**699**]: 537, 359
111	17.61	Sweroside-*O*-Ac_3-*O*-DOBA ^{L} [45,46]	619	665 ^{A1}	[**619**]: 483, 357 [**357**]: 195	621	643 ^{A2} 659 ^{A3}	[**621**]: 359
112	17.62	Desoxyoleanolic acid-*O*-hexuronide-*O*-desoxyhexoside ^{L} [63,64]	761	807 ^{A1}	[**761**]: 439	763	785 ^{A2} 801 ^{A3}	[**785**]: 617, 441 [**441**]: 423
113	17.64	Swertiamarin-*O*-Ac_2-*O*-DOBA-*O*-hexoside ^{L} [29,49]	755	801 ^{A1}	[**755**]: 593, 457, 373 [**373**]: 211	757	779 ^{A2} 795 ^{A3}	[**757**]: 375
114	17.65	Apigenin-*C*-hexoside-*O*-hexoside-*O*-Caf ^{L} [23,57,58,61]	755		[**755**] 593 [**593**] 431 [**431**] 341, 311, 313, 283	757		[**757**] 595 [**595**] 433

Table S3. Continuation

No	t_R, min	Compound [Ref.]	Negative ionization			Positive ionization		
			[M–H]⁻	Adduct ions [a]	MS/MS	[M+H]⁺	Adduct ions [a]	MS/MS
115	17.67	Sweroside-*O*-Caf [L] [53,54]	519	565 [A1]	[**519**]: 357<br>[**357**]: 195	521	543 [A2]<br>559 [A3]	[**521**] 359
116	17.85	Eustomoside-*O*-Ac3-*O*-DOBA-*O*-hexoside (gentomoside) [L,T] [29]	813	859 [A1]	[**813**]: 861, 515, 389<br>[**389**]: 227	815	837 [A2]<br>853 [A3]	[**815**] 391
117	18.08	Swertiamarin-*O*-Ac2-*O*-DOBA-*O*-hexoside [L] [29,49]	755	801 [A1]	[**755**]: 593, 457, 373<br>[**373**]: 211	757	779 [A2]<br>795 [A3]	[**757**]: 375
118	18.09	Apigenin-*C*-hexoside-*O*-hexoside-*O*-Caf [L] [23,57,58,61]	755		[**755**] 593<br>[**593**] 431<br>[**431**] 341, 311, 313, 283	757		[**757**] 595<br>[**595**] 433
120	18.10	Swertiamarin-*O*-Ac3-*O*-DOBA-*O*-hexoside-*O*-hexoside [L] [29,49]	959	1005 [A1]	[**959**]: 797, 635, 499, 373<br>[**373**]: 211	961	983 [A2]<br>999 [A3]	[**961**]: 375
121	18.11	Sweroside-*O*-Ac2-*O*-DOBA-*O*-hexoside [L] [49–51]	739	785 [A1]	[**739**]: 577, 441, 357<br>[**357**]: 195	741	763 [A2]<br>779 [A3]	[**741**]: 359
122	18.15	Acacetin-*C*-hexoside-*O*-Caf [L] [56,87]	607		[**607**] 445<br>[**445**] 355, 325, 327, 297	609		[**609**] 447
123	18.17	Desoxyoleanolic acid-*O*-hexuronide [L] [63,64]	615	661 [A1]	[**615**]: 439	617	639 [A2]<br>655 [A3]	[**617**]: 441<br>[**441**]: 423
124	18.20	Acacetin-*C*-hexoside-*O*-Caf [L] [56,87]	607		[**607**] 445<br>[**445**] 355, 325, 327, 297	609		[**609**] 447
125	18.51	Apigenin-7-*O*-glucoside [S] [57]	431		[**431**]: 269	433		[**433**]: 271
126	18.52	Sweroside-*O*-Ac3-*O*-DOBA-*O*-hexoside-*O*-hexoside [L] [51,52]	943	989 [A1]	[**943**]: 827, 619, 483, 357<br>[**357**]: 195	945	967 [A2]<br>983 [A3]	[**945**]: 359
127	18.63	Sweroside-*O*-Ac2-*O*-DOBA-*O*-hexoside [L] [49–51]	739	785 [A1]	[**739**]: 577, 441, 357<br>[**357**]: 195	741	763 [A2]<br>779 [A3]	[**741**]: 359
128	18.68	Swertiamarin-*O*-Ac3-*O*-DOBA-*O*-hexoside-*O*-hexoside [L] [29,49]	959	1005 [A1]	[**959**]: 797, 635, 499, 373<br>[**373**]: 211	961	983 [A2]<br>999 [A3]	[**961**]: 375
129	19.14	Sweroside-*O*-Ac3-*O*-DOBA-*O*-hexoside-*O*-hexoside [L] [51,52]	943	989 [A1]	[**943**]: 827, 619, 483, 357<br>[**357**]: 195	945	967 [A2]<br>983 [A3]	[**945**]: 359
130	19.16	Loganic acid-*O*-Ac3-*O*-DOBA-*O*-hexoside [L] [12,29,49]	799	845 [A1]	[**799**]: 637, 501, 375<br>[**375**]: 213	801	823 [A2]<br>839 [A3]	[**801**]: 377
131	19.18	Gelidoside (rindoside) [S] [29]	797	843 [A1]	[**797**]: 635, 499, 373<br>[**373**]: 211	799	821 [A2]<br>837 [A3]	[**799**]: 375
132	19.67	Trifloroside [S] [46]	781	827 [A1]	[**781**]: 619, 483, 357<br>[**357**]: 195	783	805 [A2]<br>821 [A3]	[**783**]: 359

Table S3. Continuation

No	t_R, min	Compound [Ref.]	Negative ionization			Positive ionization		
			[M–H]⁻	Adduct ions [a]	MS/MS	[M+H]⁺	Adduct ions [a]	MS/MS
133	19.74	Eustomoside-*O*-Ac_3-*O*-DOBA-*O*-hexoside [L] [29]	813	859 [A1]	[**813**]: 861, 515, 389 [**389**]: 227	815	837 [A2] 853 [A3]	[**815**] 391
134	21.94	Swertiamarin-*O*-Ac_3-*O*-DOBA [L] [45,46]	635	681 [A1]	[**635**]: 499, 373 [**373**]: 211	637	659 [A2] 675 [A3]	[**637**]: 375
135	22.42	Sweroside-*O*-Ac_3-*O*-DOBA [L] [45,46]	619	665 [A1]	[**619**]: 483, 357 [**357**]: 195	621	643 [A2] 659 [A3]	[**621**]: 359
136	22.67	Chrysoeriol [S] [57]	299			301		
137	22.85	Gentisin [S] [27]	257	303 [A1]		259	281 [A2]	

[a] Adduct ions was signed as follows: [A1] for $[(M–H)+HCOOH]^-$; [A2] for $[M+Na]^+$; [A3] for $[M+K]^+$. Abbreviation used: Ac—acetate, Caf—caffeoyl, DOBA—2,3-dihydroxybenzoyl, MW—molecular weight, pHBA—*p*-hydroxybenzoyl. [S] Compound identification was based on comparison with reference standard. [L] Compound identification was based on interpretation of UV and MS spectral data and comparison with literature data. [T] Tentative identification.

Table S4. Regression equations, correlation coefficients (r^2), standard deviation (S_{YX}), limits of detection (LOD), limits of quantification (LOQ) and linear ranges for 18 compounds.

**Compound** [a]	**Regression equation**	r^2	S_{YX}	**LOD (µg/mL)**	**LOQ (µg/mL)**	**Linear range (µg/mL)**
Loganic acid	$y = 0.075 \cdot x - 0.033$	0.9678	$8.31 \cdot 10^{-3}$	0.37	1.11	1.75–900.0
Swertiamarin	$y = 0.063 \cdot x - 0.023$	0.9789	$4.75 \cdot 10^{-3}$	0.25	0.75	1.00–900.0
Gelidoside	$y = 0.073 \cdot x - 0.034$	0.9581	$3.83 \cdot 10^{-3}$	0.17	0.52	1.00–900.0
Gentiopicroside	$y = 0.055 \cdot x - 0.029$	0.9294	$7.56 \cdot 10^{-3}$	0.45	1.37	1.50–900.0
Gentiopicroside-6′′-*O*-Glc	$y = 0.061 \cdot x - 0.048$	0.9377	$9.01 \cdot 10^{-3}$	0.49	1.48	1.50–900.0
Sweroside	$y = 0.067 \cdot x - 0.037$	0.9890	$7.33 \cdot 10^{-3}$	0.36	1.09	1.50–900.0
Trifloroside	$y = 0.071 \cdot x - 0.043$	0.9854	$4.27 \cdot 10^{-3}$	0.20	0.60	1.00–900.0
Isovitexin	$y = 0.054 \cdot x - 0.028$	0.9739	$5.09 \cdot 10^{-3}$	0.31	0.94	1.00–900.0
Isovitexin-2′′-*O*-Glc	$y = 0.060 \cdot x - 0.022$	0.9923	$8.07 \cdot 10^{-3}$	0.44	1.35	1.50–900.0
Saponarin	$y = 0.086 \cdot x - 0.019$	0.9781	$8.99 \cdot 10^{-3}$	0.34	1.05	1.50–900.0
Apigenin-7-*O*-Glc	$y = 0.068 \cdot x - 0.025$	0.9639	$3.78 \cdot 10^{-3}$	0.18	0.56	1.00–900.0
Isoorientin	$y = 0.033 \cdot x - 0.023$	0.9943	$6.30 \cdot 10^{-3}$	0.63	1.91	2.00–900.0
Isoorientin-2′′-*O*-Glc	$y = 0.031 \cdot x - 0.024$	0.9856	$5.42 \cdot 10^{-3}$	0.58	1.75	2.00–900.0
Isoorientin-6′′-*O*-Glc	$y = 0.083 \cdot x - 0.056$	0.9735	$8.76 \cdot 10^{-3}$	0.35	1.06	1.50–900.0
Luteolin-7-*O*-Glc	$y = 0.078 \cdot x - 0.068$	0.9867	$5.98 \cdot 10^{-3}$	0.25	0.77	1.00–900.0
Isoscoparin	$y = 0.082 \cdot x - 0.016$	0.9680	$6.34 \cdot 10^{-3}$	0.26	0.77	1.00–900.0
Mangiferin	$y = 0.045 \cdot x - 0.020$	0.9915	$8.01 \cdot 10^{-3}$	0.59	1.78	2.00–900.0
Gentioside	$y = 0.056 \cdot x - 0.013$	0.9851	$6.11 \cdot 10^{-3}$	0.36	1.09	1.50–900.0

Abbreviation used: Glc—glucose.

Table S5. Intra- and inter-day precision, repeatability, stability and recovery for 18 compounds.

Compound [a]	Precision intra-day (RSD%) n=5	Precision inter-day (RSD%) n=4	Repeatability (RSD%) n=7	Stability (RSD%) n=7	Recovery (%) n=5
Loganic acid	1.41	1.27	1.78	1.29	95.14
Swertiamarin	1.52	1.09	2.02	1.37	100.03
Gelidoside	1.33	1.33	1.74	1.88	100.78
Gentiopicroside	1.99	2.02	1.88	1.63	99.89
Gentiopicroside-6′′-*O*-Glc	2.03	1.95	1.65	1.40	96.78
Sweroside	1.89	1.77	1.19	1.03	100.95
Trifloroside	1.65	1.96	1.37	1.88	102.13
Isovitexin	1.96	1.66	1.88	2.01	101.99
Isovitexin-2′′-*O*-Glc	1.31	2.05	2.14	1.84	100.36
Saponarin	1.80	1.66	1.43	2.23	99.07
Apigenin-7-*O*-Glc	2.15	1.70	1.11	1.87	99.81
Isoorientin	2.21	1.82	2.08	1.72	94.95
Isoorientin-2′′-*O*-Glc	1.73	1.59	1.59	1.39	103.56
Isoorientin-6′′-*O*-Glc	1.98	1.36	1.49	2.12	102.14
Luteolin-7-*O*-Glc	1.62	1.28	1.74	1.06	100.78
Isoscoparin	1.36	1.94	1.89	1.85	96.34
Mangiferin	1.47	1.97	1.64	1.59	97.21
Gentioside	1.54	2.15	1.32	1.91	95.29

Abbreviation used: Glc—glucose.

Table S6. Content of selected phenolic compounds in dry extracts of gentian herbs and roots [a], mg/g of dry extract weight (±S.D.).

Compound	GAS	GCR	GGE	GPA	GPN	GSE
Gentian herb extracts						
Isovitexin	3.15 ± 0.06	10.44 ± 0.19	4.09 ± 0.08	1.91 ± 0.04	2.94 ± 0.06	14.46 ± 0.28
Isovitexin-2′′-*O*-Glc	6.42 ± 0.12	10.80 ± 0.21	5.42 ± 0.11	8.23 ± 0.16	5.20 ± 0.10	28.12 ± 0.56
Saponarin	4.41 ± 0.08	6.11 ± 0.12	0.04 ± 0.00	5.65 ± 0.11	4.46 ± 0.09	33.32 ± 0.65
Apigenin-7-*O*-Glc	8.82 ± 0.17	2.16 ± 0.04	2.54 ± 0.05	2.45 ± 0.05	1.41 ± 0.03	0.40 ± 0.01
Isoorientin	64.09 ± 1.28	21.99 ± 0.43	131.03 ± 2.62	23.89 ± 0.47	89.29 ± 1.78	68.88 ± 1.40
Isoorientin-2′′-*O*-Glc	142.17 ± 2.85	72.23 ± 1.44	59.14 ± 1.18	50.65 ± 1.01	19.03 ± 0.38	80.48 ± 1.61
Isoorientin-6′′-*O*-Glc	0.00	0.00	12.23 ± 0.24	0.00	0.00	33.50 ± 0.67
Luteolin-7-*O*-Glc	4.52 ± 0.09	0.84 ± 0.02	3.04 ± 0.06	4.83 ± 0.09	7.51 ± 0.15	9.57 ± 0.19
Isoscoparin	0.00	0.00	4.33 ± 0.08	0.00	8.15 ± 0.16	1.48 ± 0.03
Mangiferin	59.43 ± 1.18	26.46 ± 0.52	0.00	17.41 ± 0.34	12.73 ± 0.25	0.00
Total phenolic compounds	293.01	151.03	221.86	115.02	150.72	270.21
Gentian root extracts						
Isoorientin-2′′-*O*-Glc	17.28 ± 0.34	0.00	0.00	0.00	0.00	0.00
Gentioside	2.93 ± 0.06	0.00	0.00	0.00	0.00	0.00
Total phenolic compounds	20.21	0.00	0.00	0.00	0.00	0.00

[a] Gentian species: GAS—*Gentiana asclepiadea*, GCR—*Gentiana cruciata*, GGE—*Gentiana gelida*, GPA—*Gentiana paradoxa*, GPN—*Gentiana pneumonanthe*, GSE—*Gentiana septemfida*. "tr."—trace content (<LOQ). Abbreviation used: Glc—glucose.

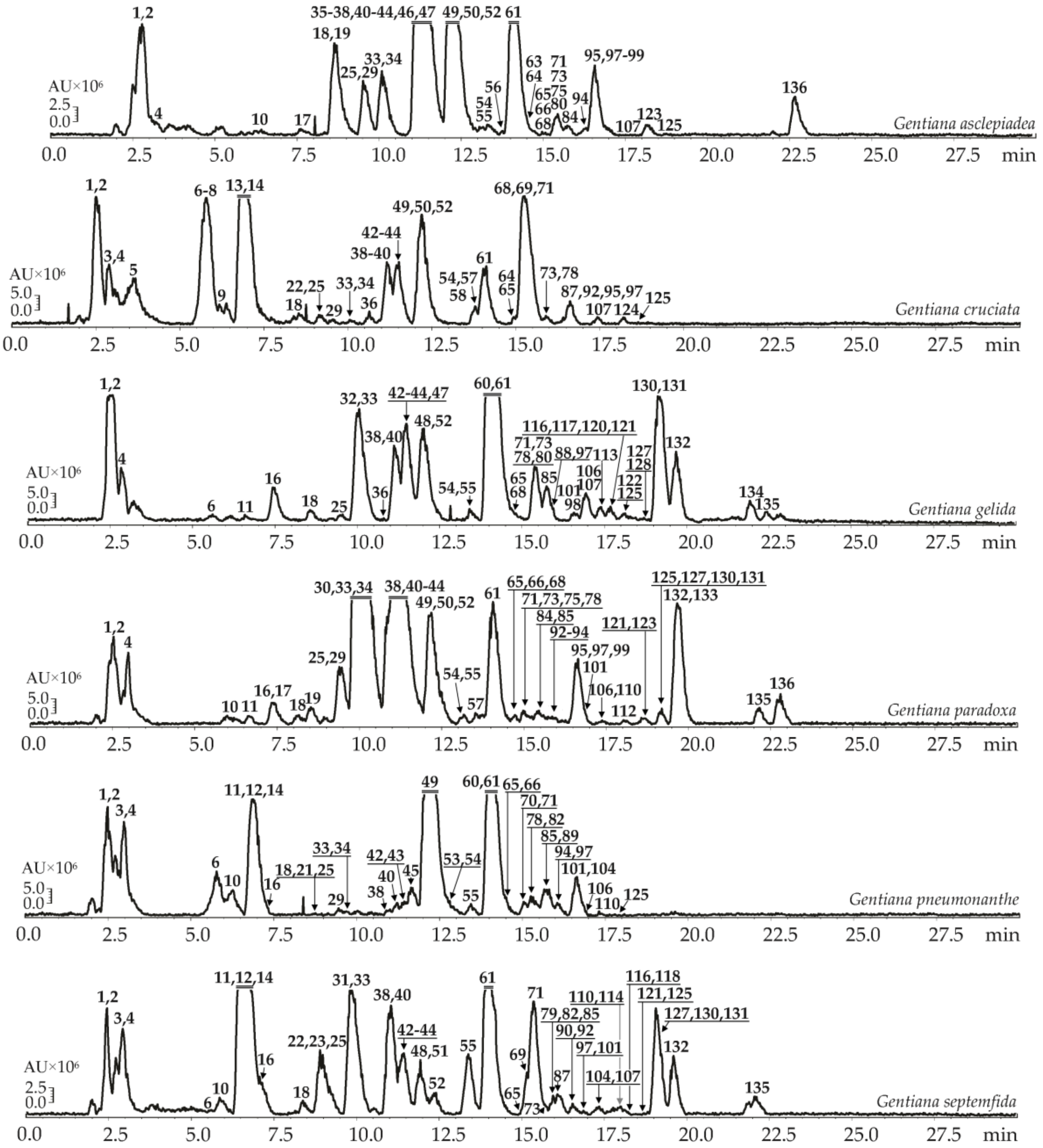

Figure S1. High-Performance Liquid Chromatography with Electrospray Ionization Triple Quadrupole Mass Spectrometric Detection (HPLC-ESI-QQQ-MS) chromatogram in base peak chromatogram mode (BPC mode, negative ionization) of six *Gentiana* herb and root extracts. Compounds are numbered as listed in Table 1.

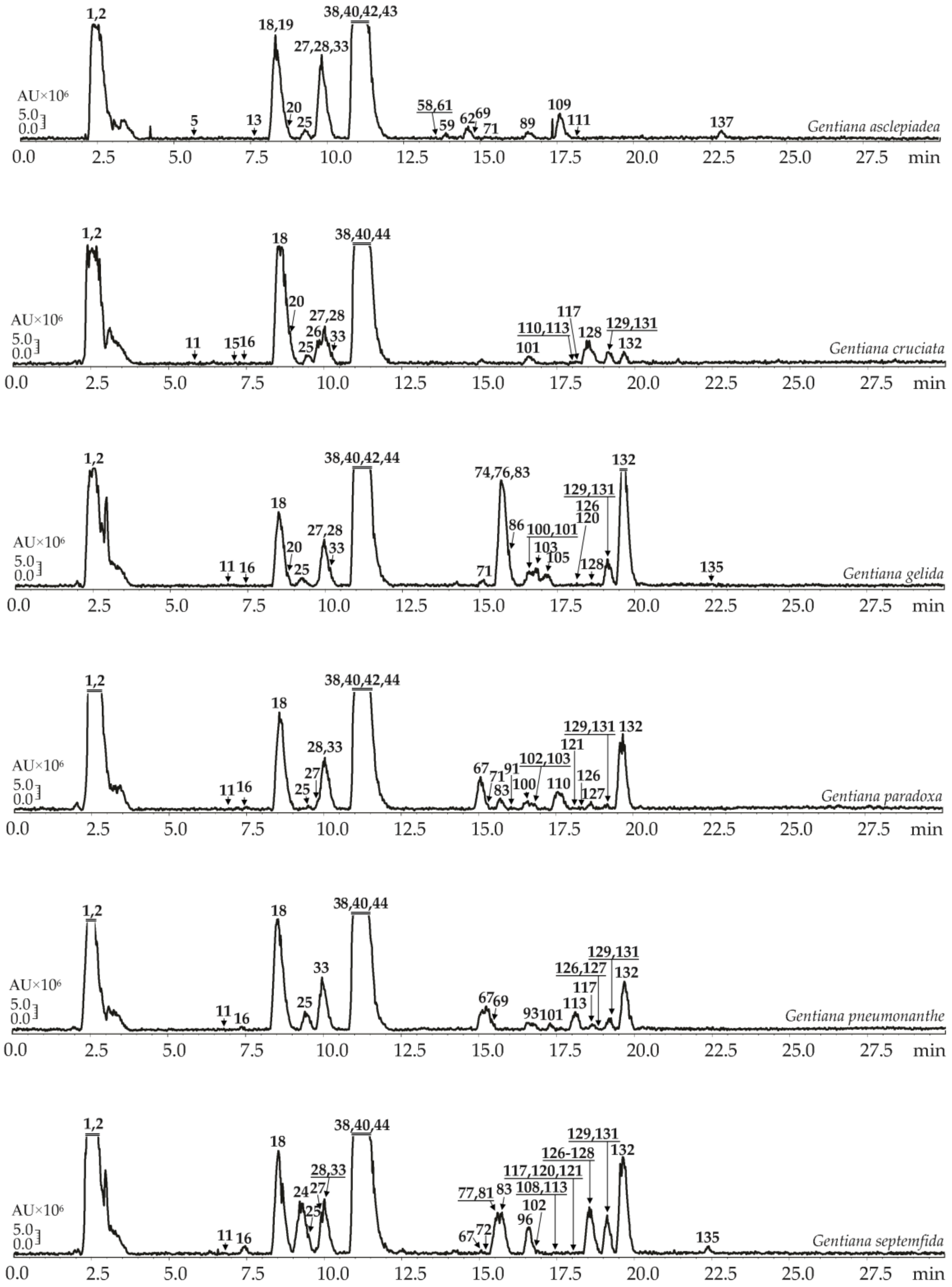

Figure S1. *Continuation.*

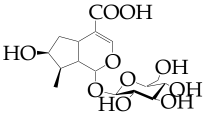

Loganic acid (**9**)

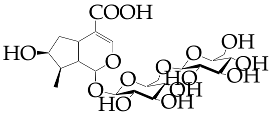

Loganic acid-6′-*O*-glucoside (**15**)

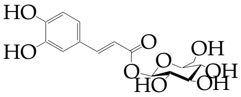

1-*O*-Caffeoyl-glucose (**21**)

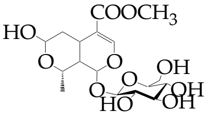

Morroniside (**23**)

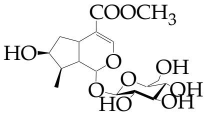

Loganin (**25**)

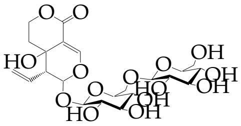

Swertiamarin-6′-*O*-glucoside (**26**)

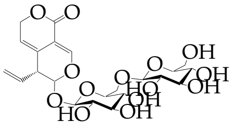

Gentiopicroside-6′-*O*-glucoside (**27**)

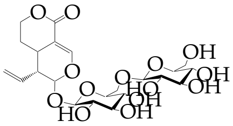

Sweroside-6′-O-glucoside (**28**)

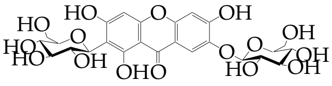

Mangiferin-7-*O*-glucoside (**49**)

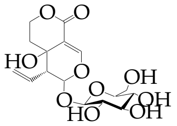

Swertiamarin (**33**)

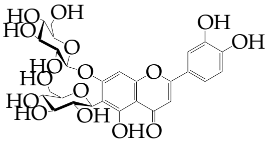

Isoorientin-7-*O*-glucoside (**35**)

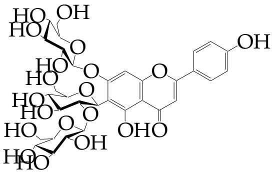

Isovitexin-7,2′′-*O*-glucoside (**37**)

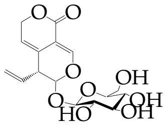

Gentiopicroside (**38**)

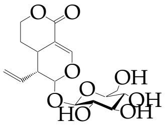

Sweroside (**40**)

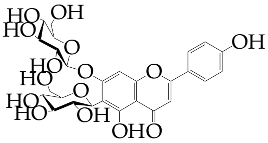

Isovitexin-7-*O*-glucoside (**42**)

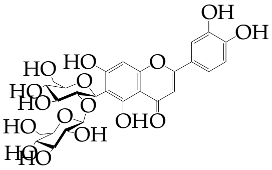

Isoorientin-2′′-*O*-glucoside (**43**)

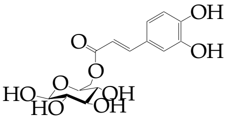

6-*O*-Caffeoyl-glucose (**45**)

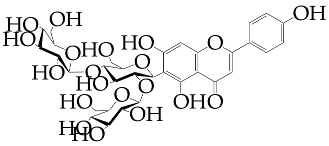

Isovitexin-2′′,4′′-*O*-glucoside (**47**)

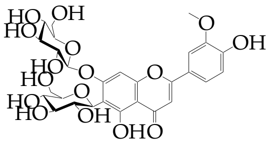

Isoscoparin-7-*O*-glucoside (**48**)

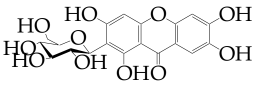

Mangiferin (**49**)

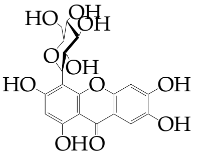

Isomangiferin (**50**)

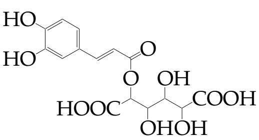

2-*O*-Caffeoyl-glucaric acid (**51**)

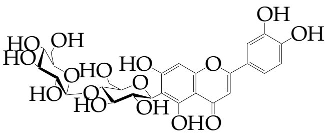

Isoorientin-4′′-*O*-glucoside (**52**)

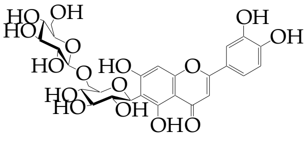

Isoorientin-6′′-*O*-glucoside (**54**)

Figure S2. Structures of reference compounds (numbered accordingly to Table 1).

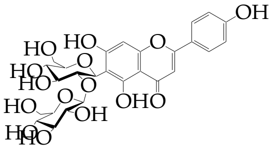

Isovitexin-2′′-*O*-glucoside (**55**)

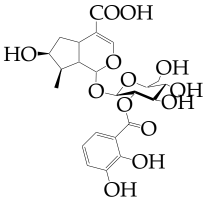

Algidiside I (**58**)

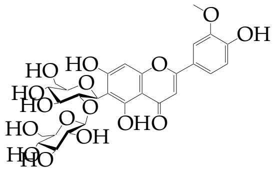

Isoscoparin-2′′-*O*-glucoside (**60**)

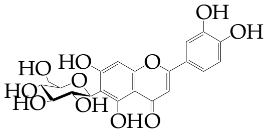

Isoorientin (**61**)

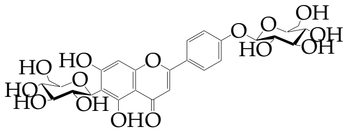

Isovitexin-4′-O-glucoside (**64**)

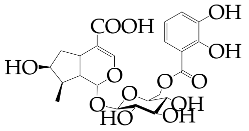

Algidiside II (**69**)

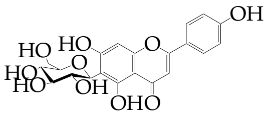

Isovitexin (**71**)

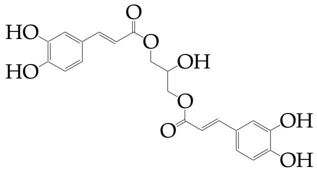

1,3-Di-*O*-Caffeoyl-glycerol (**79**)

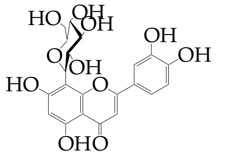

Orientin (**80**)

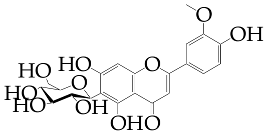

Isoscoparin (**85**)

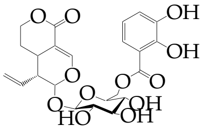

6′-*O*-(2,3-Dioxybenzoyl)-sweroside (**83**)

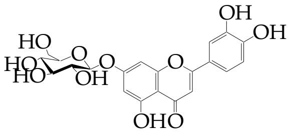

Luteolin-7-*O*-glucoside (**98**)

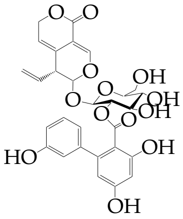

Amarogentin (**105**)

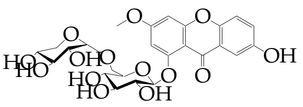

Gentioside (**109**)

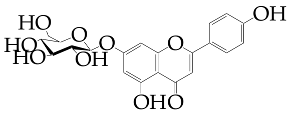

Apigenin-7-*O*-glucoside (**125**)

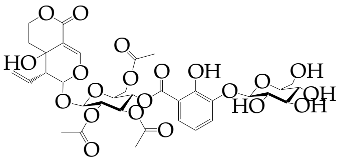

Gelidoside (rindoside; **131**)

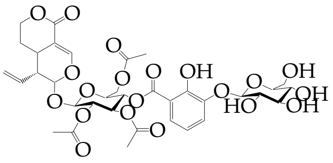

Trofloroside (**132**)

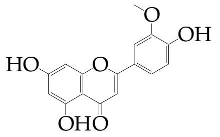

Chrysoeriol (**136**)

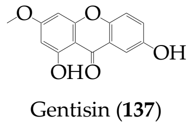

Gentisin (**137**)

Figure S2. *Continuation.*

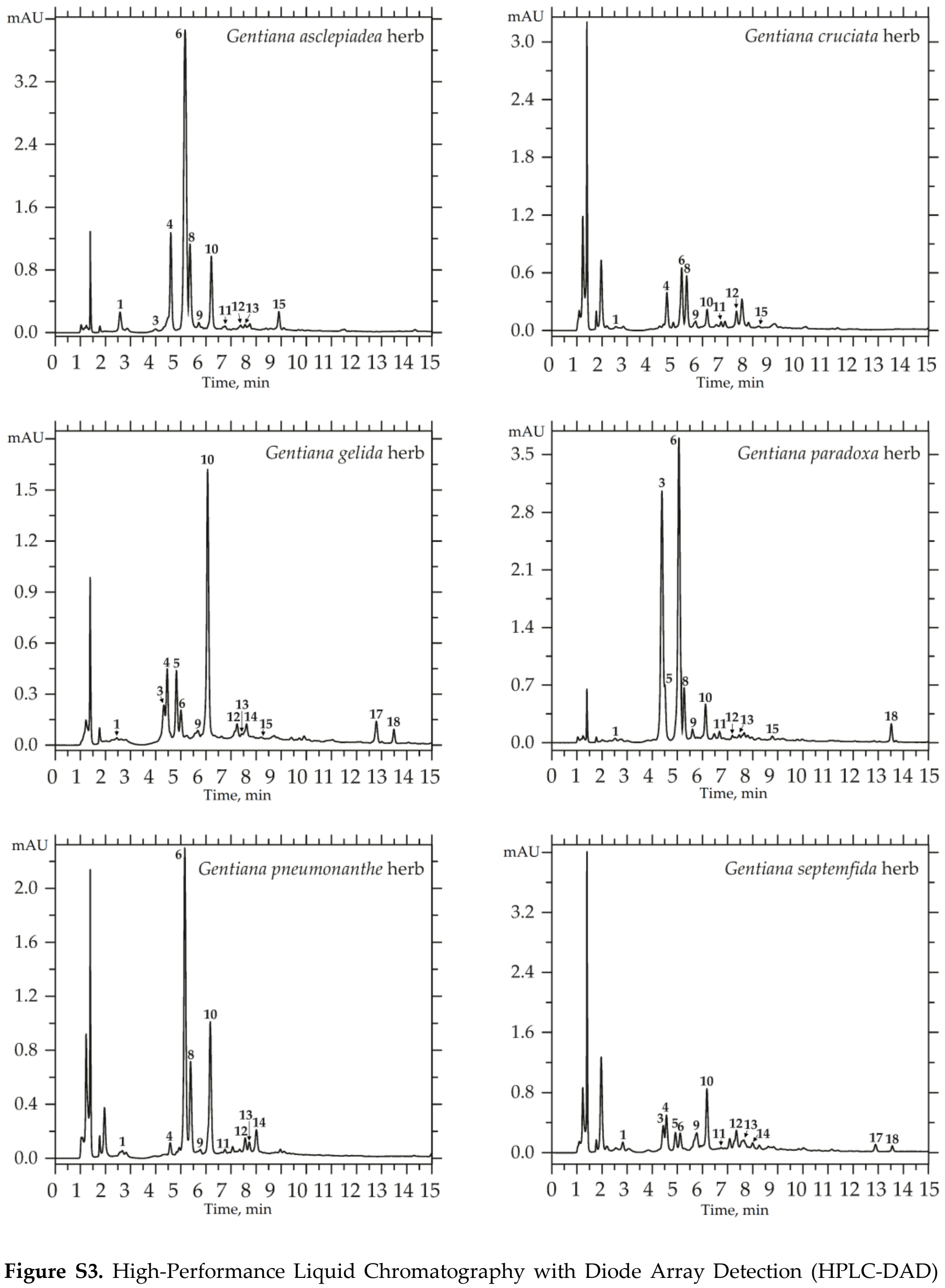

Figure S3. High-Performance Liquid Chromatography with Diode Array Detection (HPLC-DAD) chromatograms of gentian herb and roots extracts at 210 nm. Compounds are numbered as follows: **1**—loganic acid; **2**—gentiopicroside-6′′-*O*-glucoside; **3**—swertiamarin; **4**—isoorientin-2′′-*O*-glucoside; **5**—isoorientin-6′′-*O*-glucoside; **6**—gentiopicroside; **7**—sweroside; **8**—mangiferin; **9**—isovitexin-2′′-*O*-glucoside; **10**—isoorientin; **11**—saponarin; **12**—isovitexin; **13**—luteolin-7-*O*-glucoside; **14**—isoscoparin; **15**—apigenin-7-*O*-glucoside; **16**—gentioside; **17**—gelidoside (rindoside); **18**—trifloroside.

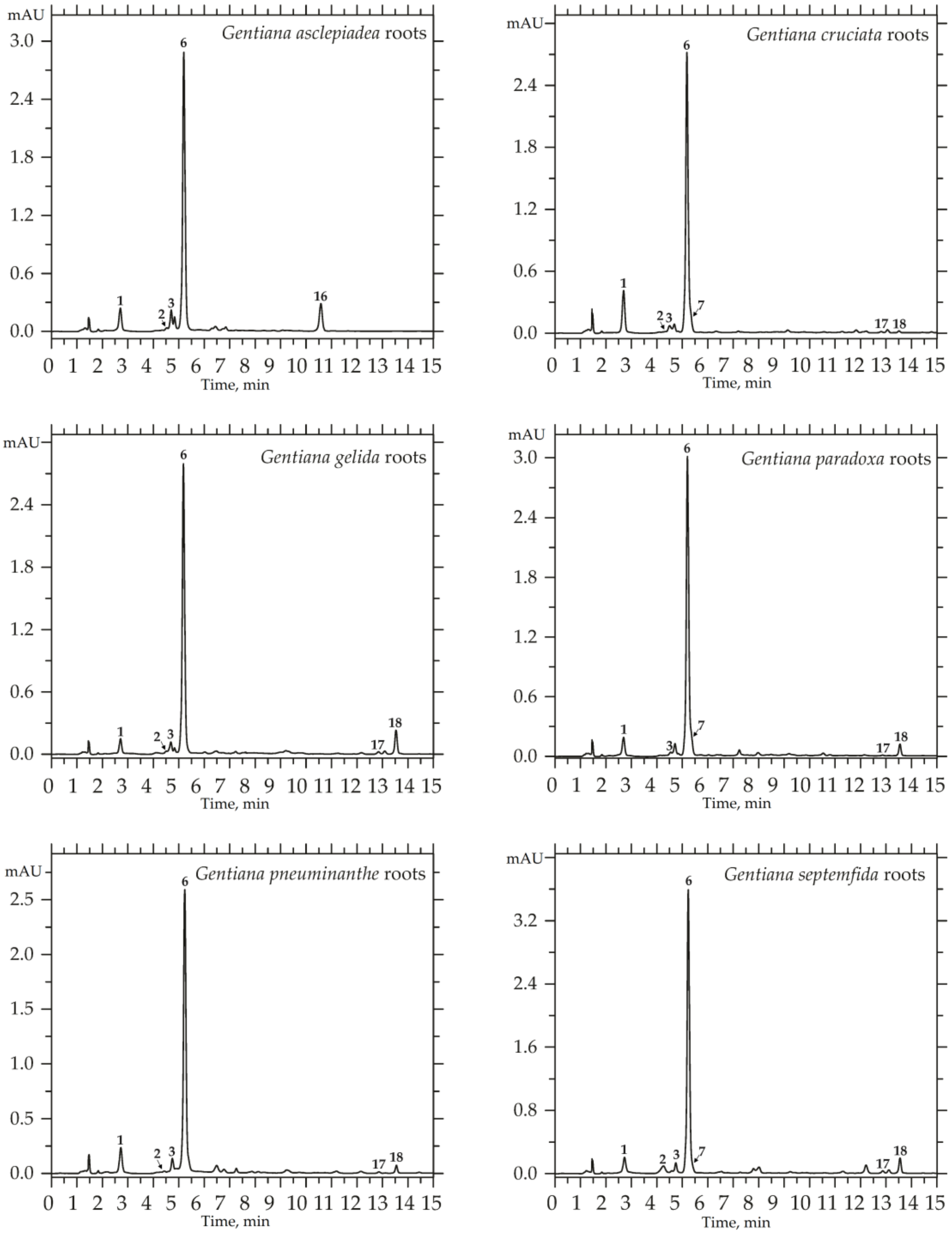

Figure S3. *Continuation*.
